# Chromatin topology is coupled to Polycomb group protein subnuclear organization

**DOI:** 10.1038/ncomms10291

**Published:** 2016-01-13

**Authors:** Ajazul H. Wani, Alistair N. Boettiger, Patrick Schorderet, Ayla Ergun, Christine Münger, Ruslan I. Sadreyev, Xiaowei Zhuang, Robert E. Kingston, Nicole J. Francis

**Affiliations:** 1 Department of Molecular Biology, Massachusetts General Hospital, Boston, Massachusetts, USA; 2 Department of Genetics, Harvard Medical School, Boston, Massachusetts, USA; 3 Howard Hughes Medical Institute, Harvard University Cambridge, Cambridge, Massachusetts 02138, USA; 4 Department of Chemistry and Chemical Biology, Harvard University, Cambridge, Massachusetts, USA; 5 Department of Physics, Harvard University, Cambridge, Massachusetts 02138, USA; 6 Institut de recherches cliniques de Montréal, Montréal, Québec, Canada; 7 Department of Pathology, Massachusetts General Hospital and Harvard Medical School, Boston, Massachusetts, USA; 8 Département de biochimie et medécine moléculaire, Université de Montréal, Montréal, Québec, Canada

## Abstract

The genomes of metazoa are organized at multiple scales. Many proteins that regulate genome architecture, including Polycomb group (PcG) proteins, form subnuclear structures. Deciphering mechanistic links between protein organization and chromatin architecture requires precise description and mechanistic perturbations of both. Using super-resolution microscopy, here we show that PcG proteins are organized into hundreds of nanoscale protein clusters. We manipulated PcG clusters by disrupting the polymerization activity of the sterile alpha motif (SAM) of the PcG protein Polyhomeotic (Ph) or by increasing Ph levels. Ph with mutant SAM disrupts clustering of endogenous PcG complexes and chromatin interactions while elevating Ph level increases cluster number and chromatin interactions. These effects can be captured by molecular simulations based on a previously described chromatin polymer model. Both perturbations also alter gene expression. Organization of PcG proteins into small, abundant clusters on chromatin through Ph SAM polymerization activity may shape genome architecture through chromatin interactions.

## Abstract (web summary)

Polycomb Group (PcG) proteins regulate gene expression and genome architecture. Using super-resolution microscopy and molecular simulations, Wani *et al*. describe the organization of PcG proteins into hundreds of nano-scale protein clusters and suggest these clusters shape genome architecture.

## Graphical abstract

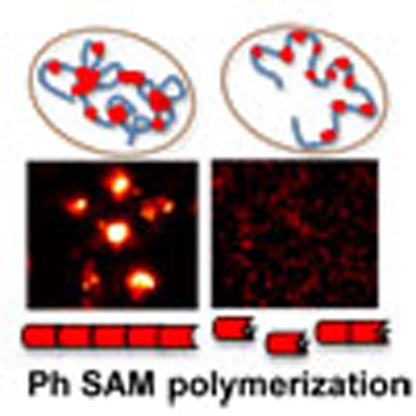

A major question in eukaryotic biology is how the gene regulatory machinery and its chromatin substrate are organized in the nucleus^1^,^2^. High resolution descriptions demonstrate that chromatin in eukaryotic nuclei is organized at multiple scales, from individual nucleosomes to specific loops between regulatory sequences, to the folding of large genomic regions into topological domains and segregation of whole chromosomes into territories^3^,^4^,^5^,^6^. Organization of chromatin at all scales must be shaped by chromatin proteins, but we are just beginning to understand how specific chromatin proteins contribute to observed configurations^5^,^7^.

Polycomb group (PcG) proteins are essential developmental regulators that assemble into multiprotein complexes that modify chromatin to repress gene expression^8^. Part of this regulation is believed to occur at the level of chromatin organization. PcG complexes can compact chromatin^9^, and PcG-dependent compaction has been observed at some PcG-regulated loci^10^. Some PcG protein-bound regions of chromatin are organized into distinct topological domains^11^,^12^, and depleting PcG proteins can decrease interactions among PcG-bound sites inside a PcG domain^13^. Interactions among PcG domains have also been described^14^,^15^,^16^, and suggested to have a role for PcG proteins in stress-induced genome reorganization^17^. Finally, in *Drosophila* embryos and cell lines, and in mammalian cells, members of a key PcG complex, Polycomb Repressive Complex 1 (PRC1), have been observed to form a variable number of PcG ‘bodies' or ‘foci'^1^,^3^,^18^ (Fig. 1a,c), which have been suggested to bring PcG-regulated genes together^15^,^17^,^19^.

Here we combine super-resolution microscopy, chromosome conformation capture, chromatin immunoprecipitation, RNA-seq and molecular simulation to analyse PcG protein subnuclear organization and its impact on chromatin topology and gene expression. We find that PcG proteins form hundreds of small protein clusters in nuclei, distinct from the large PcG bodies present in just a few copies per cell that have been the focus of previous investigations. Our results implicate the polymerization activity of the Polyhomeotic sterile alpha motif (Ph SAM)^20^ in PcG clustering and further suggest that PcG clustering influences chromatin interactions at multiple scales. We suggest that the nanoscale organization of PcG proteins into small, abundant clusters on chromatin through the polymerization activity of Ph SAM shapes genome architecture by mediating numerous long-range chromatin interactions.

## Results

### PcG proteins form hundreds of nanoscale clusters in nuclei

**Figure 1:**
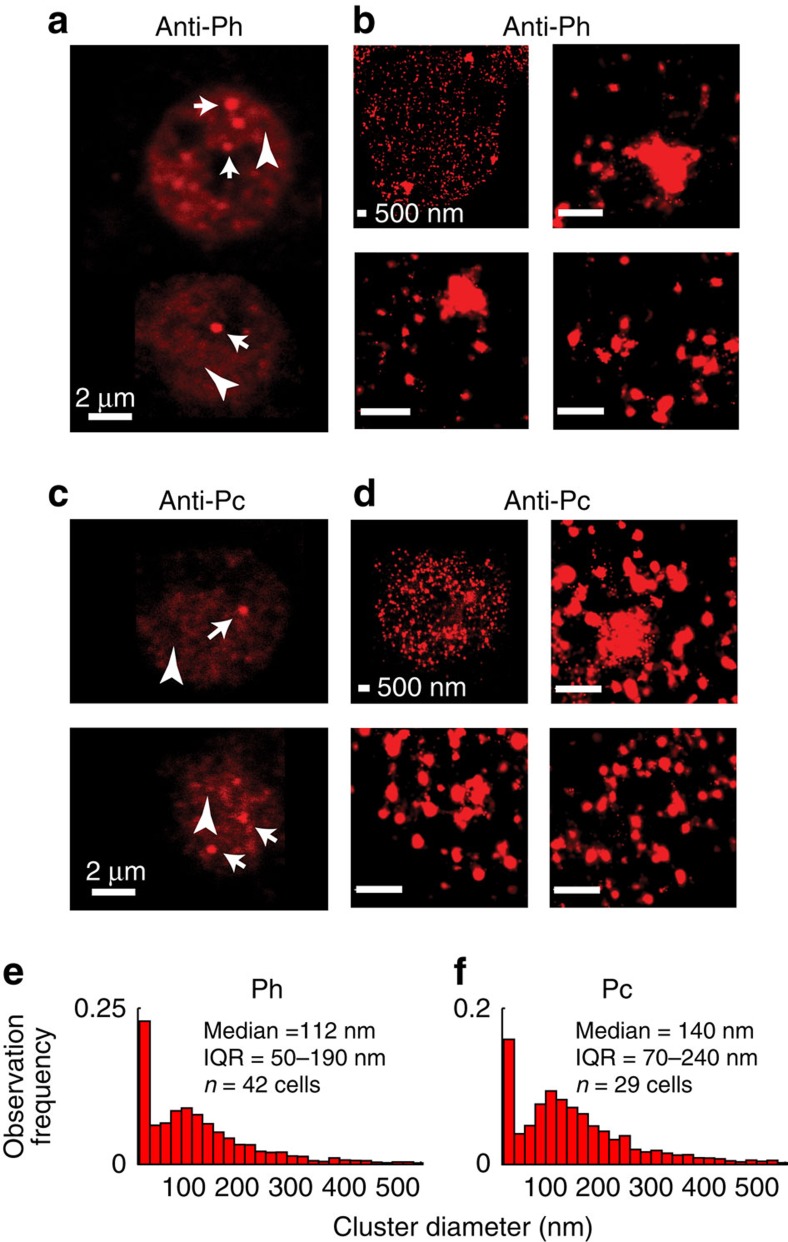
PRC1 has a multi-scale subnuclear organization. (**a**,**c**) Confocal images showing Ph (**a**) or Pc (**c**) foci (arrows) and diffuse staining (arrow heads) outside foci in *Drosophila* S2 cells immunostained with anti-Ph (**a**) or anti-Pc (**c**) antibodies. (**b**,**d**) STORM images of *Drosophila* S2 cells. Top left panels show whole nuclei and the other three panels higher magnification views of clusters of different sizes. (**e**,**f**) Distribution of cluster sizes of Ph (**e**) or Pc (**f**) subnuclear clusters observed by STORM imaging. IQR, interquartile range (value of the 25% percentile to the 75% percentile). Scale bars in **a** and **c** are 2 μm and are 500 nm in **b** and **d**. At least two replicate experiments were carried out; differences between replicates were not detected and cells from replicates are pooled.

To understand PcG protein subnuclear organization, we used stochastic optical reconstruction microscopy (STORM)^21^,^22^ in *Drosophila* S2 cells to localize individual antibodies bound to PRC1 components (Supplementary Fig. 1). STORM reveals that the PRC1 components Polycomb (Pc) and Ph are distributed throughout the nucleus in clusters ranging from ∼30 nm to >700 nm in diameter (Fig. 1b,d). The largest clusters are presumably the PcG bodies visible by conventional images (Fig. 1a,c), while many of the smaller clusters are too closely distributed to be resolved by conventional imaging. Clusters of Ph and Pc were quantified by plotting the fraction of total localizations in clusters of different sizes. As we expect the number of molecules in a cluster to scale with the third power of the cluster diameter, plotting the distribution of cluster sizes over emphasizes small clusters, which represent a very small fraction of the total Ph in the cell, but constitute a disproportionate amount of the total clusters. Weighting clusters by the number of localizations detected, the median diameter for Ph was 110 nm, and for Pc, 140 nm (Fig. 1e,f). Re-analysis of our STORM images using only half of the single-molecule localization events yielded a size distribution of clusters that is very similar to that in Fig. 1 (Supplementary Fig. 2), indicating that our sampling density is sufficiently high for a robust determination of the cluster sizes.

An average of ∼200 clusters per cell (larger than our 30 nm resolution limit) was counted in S2 cells (median 143, interquartile range 82–244, *n*=42 cells). Because we image an optical section of 800–1,000 nm, we predict that the actual number of clusters per nucleus is ∼600–800. Thus, STORM reveals that PcG proteins are organized into an extensive array of small clusters whose size and distribution could not be detected in the previous work^1^,^19^. While large PcG bodies are rare, clustering of PcG proteins is pervasive in nuclei.

### Ph SAM polymerization activity drives PcG clustering

Ph contains a highly conserved SAM in its C-terminal region. This domain can form head to tail polymers *in vitro*^20^ and is essential for Ph-mediated gene silencing, development and growth control in *Drosophila*^23^,^24^. The Ph SAM is also implicated in the subnuclear organization of mammalian PcG proteins and regulation of gene expression^25^. We hypothesized that the polymerization activity of the Ph SAM could produce the observed wide range of PcG cluster sizes. Ph SAM contains two polymerization interfaces, the end helix (EH) and mid-loop (ML). Mutation of both interfaces eliminates SAM–SAM interactions, while mutation of a single interface creates a SAM that can bind to wild-type Ph (Ph-WT) SAM through its intact interface but cannot form polymers^20^. These mutations have previously been shown to have dominant negative function, and were used to show that Ph SAM polymerization capacity is important for maintaining *Hox* gene repression and for normal development in *Drosophila*^23^,^24^. To test the possible contribution of Ph SAM polymerization to PcG protein clustering, we created stable S2 cell lines that express (FLAG-tagged) Ph-WT or Ph with mutations that disrupt one polymerization interface (Ph-ML; ref. 20; Supplementary Fig. 3). These Ph transgenes were expressed in the context of endogenous Ph so that both transgenic and endogenous Ph are present in all of the experiments described below. Another PcG protein, SCM, also has a SAM that can form co-polymers with Ph SAM, preferentially interacting with the ML interface of Ph SAM (ref. 26). SCM is a non-stoichiometric member of PRC1 *in vivo*^27^, and its assembly into recombinant PRC1 depends on Ph (ref. 28). Interfering with Ph SAM polymerization activity in cells is thus expected to affect both Ph–Ph and Ph-SCM interactions, although it is expected that Ph–Ph interactions are more prevalent.

**Figure 2:**
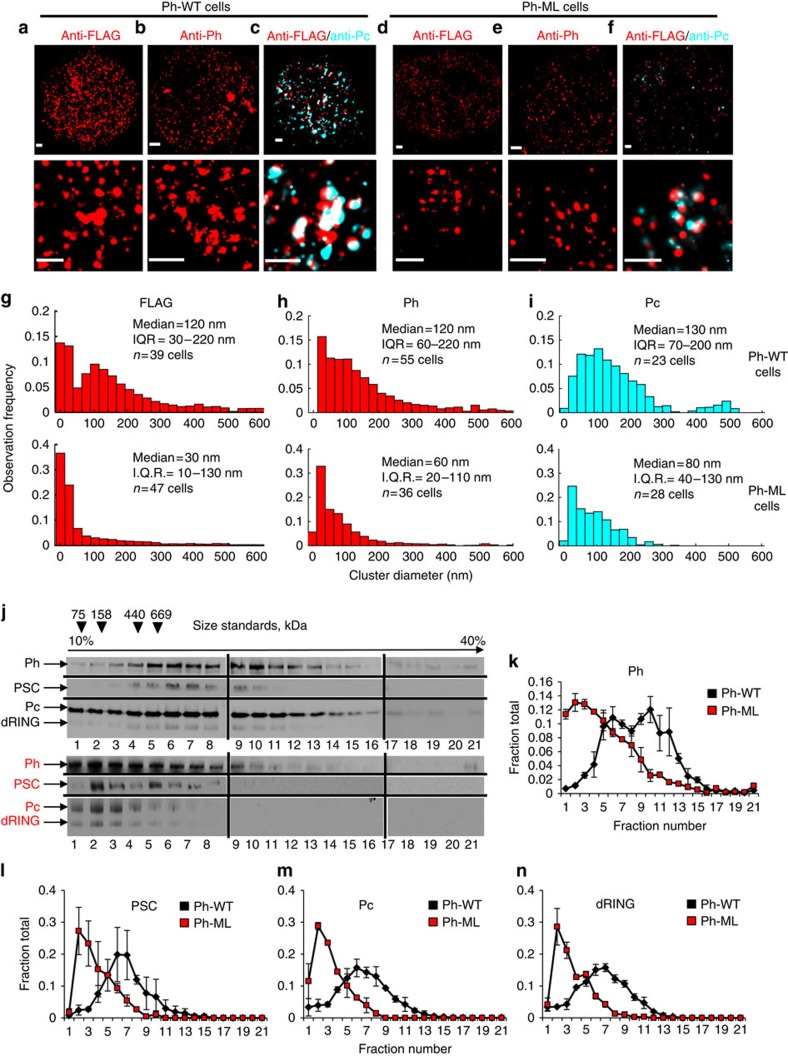
PRC1 clustering depends on Ph SAM polymerization activity. *Drosophila* S2 cell lines expressing either Ph-WT (**a**–**c**) or Ph-ML (**d**–**f**) were stained with the indicated antibodies and imaged by STORM. Top panels show whole nuclei. Scale bars, 500 nm. (**g**–**i**) Histograms show the distribution of cluster sizes observed by STORM. At least two replicate experiments were carried out; differences between replicates were not detected and cells from replicates are pooled. (**j**) Western blots showing sedimentation profiles of Ph-WT (top) or Ph-ML (bottom) PcG complexes purified from nuclear extracts. (**k**–**n**) Graphs show quantification of the distribution of each of the four core subunits of PRC1 in gradients. Values are the average of two different experiments and error bars show the data range.

We imaged S2 cells expressing ectopic, FLAG-tagged Ph-WT and Ph-ML, in addition to endogenous Ph, selecting cells of either type with similar brightness of bulk anti-FLAG fluorescence. We find that Ph-WT has a clustered organization with size distribution similar to that observed for endogenous Ph (Fig. 2a,g). However, we observe a dramatic change in the organization of Ph-ML (Fig. 2) with a much smaller median cluster size of 30 nm. We note that when the cluster size is ∼30 nm, comparable to our image resolution, we cannot exclude the possibility that these apparent clusters of localizations correspond to single molecules of Ph-ML bound by antibodies. A few larger clusters are still observed, which likely reflect binding of Ph-ML to clusters of endogenous, Ph-WT (Fig. 5). This distribution is consistent with Ph-ML being unable to form clusters effectively, suggesting SAM polymerization activity is essential for this activity.

### Ph SAM mutations disrupt endogenous PcG clusters

To determine how expression of Ph-WT or Ph-ML affects the nuclear organization of endogenous Ph, antibodies to Ph were used to detect both endogenous and ectopic Ph. In Ph-WT-expressing cells (Fig. 2b), the cluster-size distribution of total Ph is nearly identical to that of FLAG-Ph, with a 120-nm median cluster diameter (Fig. 2h), and only slightly larger than the median diameter in control S2 cells not expressing FLAG-tagged Ph-WT. Because the total Ph level in Ph-WT cells is at least twofold higher than in control S2 cells (Supplementary Fig. 3), these data suggested that Ph-WT cells would contain more clusters. This is indeed the case, with an average of ∼500 clusters larger than our resolution limit counted in Ph-WT cells, as opposed to ∼200 in the control S2 cells. In cells expressing Ph-ML (Fig. 2e), the median cluster diameter of Ph (wild type and Ph-ML) drops to 60 nm (Fig. 2h). The larger diameter of clusters detected with anti-Ph than anti-FLAG is consistent with the ability of Ph-ML to bind to Ph but not to another Ph-ML. This observation is further supported by analysing the effect of Ph-ML concentration on cluster diameter (Fig. 5a–c). We conclude that multi-scale clustering of Ph depends on the oligomerization capacity of its SAM, and that Ph-ML functions as a dominant negative to disrupt endogenous Ph clusters.

To determine whether expression of Ph-ML affects the distribution of PRC1 or just Ph, we analysed clusters containing the PRC1 member Pc in cells expressing Ph-WT or Ph-ML (Fig. 2c,f). We find that 50–60% of Ph-WT localizations detected by the antibody against the FLAG-tag colocalize with Pc localizations detected by the antibody against Pc, and 40–60% of Pc localizations colocalize with Ph-WT localizations. Pc clusters which contained Ph-WT have a similar size distribution as that of Ph-WT clusters (compare Fig. 2i with Fig. 2g). In cells expressing Ph-ML, Pc clusters associated with Ph-ML are smaller, with a median cluster diameter of 80 nm (Fig. 2i). Very few large Pc clusters (>300 nm diameter) are observed in these cells. As a control for our ability to detect colocalization, we analysed cells stained with both anti-FLAG and anti-Ph (Supplementary Fig. 4). We find a high degree of overlap both in Ph-WT cells (75–80%) and in Ph-ML cells (∼90%). This substantial overlap suggests that the efficiency of antibody binding in our assay is high. We conclude that disrupting the Ph SAM polymerization activity impacts Pc clustering, suggesting the Ph SAM regulates the organization of PRC1 into multi-scale clusters.

### Ph SAM mutations alter the sedimentation of Ph complexes

Previous studies indicate that interactions among PRC1 components do not depend on Ph SAM (ref. 29). However, to confirm that disrupting Ph polymerization activity does not impair complex formation in S2 cells, Ph-containing complexes were purified from nuclear extracts of cells expressing Ph-WT, Ph-ML or Ph-EH (mutation in the other polymerization interface). Mass spectrometry analysis of these complexes confirms that they contain similar levels of PRC1 subunits (Supplementary Fig. 5a,b). We also compared the abilities of Ph-WT, Ph-ML, and Ph delta SAM to assemble with the other core PRC1 subunits in Sf9 cells and find that all three form PRC1 that is stable under our purification conditions (2M KCl wash; Supplementary Fig. 5c). Thus, functional effects of Ph-ML are unlikely due to its impaired assembly into PRC1.

The Ph SAM-dependent clustering of PRC1 components suggests that PRC1 is present in oligomeric forms that are disrupted by expression of Ph-ML. To test this idea, Ph-containing complexes were purified from nuclear extracts of cells expressing Ph-WT or Ph-ML and analysed by glycerol gradient sedimentation. Levels of core PRC1 subunits were analysed in gradient fractions by Western blotting and have similar sedimentation profiles. The peak of Ph-WT complexes sediments more rapidly than a 669-kDa molecular weight standard with PRC1 components detected in a long tail through the bottom of the gradient. The peak of Ph-ML complexes is shifted towards the top of the gradient, above the 669-kDa standard (Fig. 2j–n). We also tested complexes formed by Ph with mutations in the EH interface and find that they behave very similarly (Supplementary Fig. 6). These results are consistent with Ph-WT complexes existing in oligomeric forms that are disrupted by mutations in the SAM. They are also consistent with the effect of Ph SAM on sedimentation of PRC1 from mammalian cells^25^.

### PcG clustering affects chromatin interactions in the *BX-C*

To test whether PcG protein clustering affects chromatin organization, we used the ‘4C' derivative of the chromosome conformation capture methodology^30^. When coupled with next generation sequencing (4C-seq)^31^, 4C identifies sequences genome wide that come into proximity to a specific ‘viewpoint' sequence. We used three viewpoint sequences in the *Bithorax-Complex* (*BX-C) Hox* gene cluster (which is composed of well-characterized PcG chromatin topological domains) to query chromatin interactions. 4C-seq was carried out in duplicate with each of the three viewpoint sequences in normal S2 cells, and those expressing either Ph-WT or polymerization defective Ph-ML. 4C-seq reads were mapped to the genome^32^; the overall distributions and numbers of reads were similar across the three cell lines (Supplementary Fig. 7).

**Figure 3:**
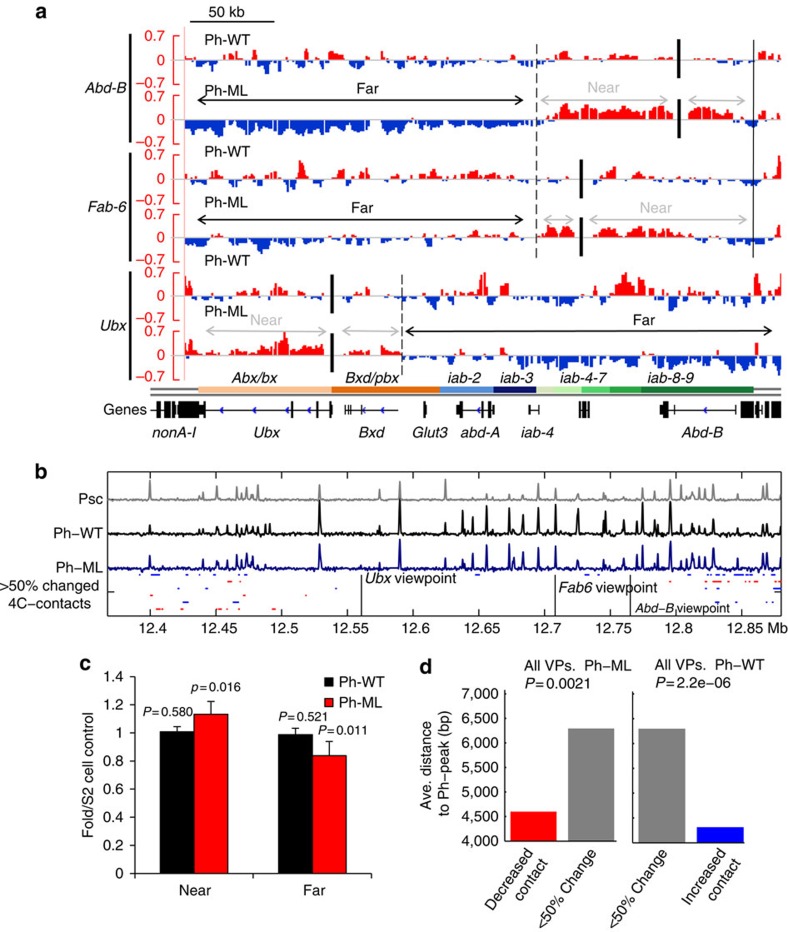
Ph SAM-dependent effects on chromatin organization in the *Bithorax-Complex* of *Hox* genes. (**a**) Chromatin interactions in the *BX-C* as determined by 4C-seq. Tracks show the average (of duplicates) ratio of reads in Ph-WT or Ph-ML expressing cells to those in control S2 cells, minus one so that regions with increased reads are positive and those with decreased reads are negative. Vertical black bars indicate the viewpoint positions. Coloured bars at the bottom of the panel indicate the *BX-C* regulatory regions. Green elements (*iab4-9*) are implicated in regulating *Abd-B*, blue (*iab2-3*) in regulating *abd-A* and orange in regulating *Ubx* (refs 66, 67). Dashed lines indicate the boundaries of ‘near' and ‘far' regions used in the analysis in **c**, which are demarcated with grey and black arrows respectively. For the *Ubx* viewpoint, the boundary of the ‘near' region is set at the start of the *bxd* non-coding transcript. (**b**) ChIP-seq traces of binding of PSC, Ph-WT and Ph-ML across *BX-C* compared with areas of >50% increased 4C contacts relative to S2 in Ph-WT cells (blue) or decreased Ph-contacts in Ph-ML cells (red). (**c**) Quantification of near and far contacts in the *BX-C*. Contacts were normalized to the S2 cell control and these normalized data averaged overall three viewpoints from two experiments. Error bars are s.d. and *P* values are for two-tailed, Student's *t*-tests. Data distribution did not violate the assumption of normality by Anderson Darling tests. (**d**) Quantification of distance from regions with >50% change in interaction frequency to the center of the nearest Ph peak. See also Supplementary Figs 7–9.

Because Ph-ML is expressed in the context of endogenous Ph in these experiments, and because expression is not uniform in all cells (see below), the effect of disrupting PcG clustering is likely to be underestimated in assays of the whole-cell population. Therefore, to highlight differences among the different cell types, we normalized the reads in the *BX-C* from Ph-WT or Ph-ML cells to those from S2 cells (Fig. 3a). In Ph-WT-expressing cells, segments with increased and decreased interactions relative to S2 cells are interspersed throughout the domain. In contrast, in cells expressing Ph-ML, the viewpoints have decreased interactions with more distant regions of the cluster as compared with S2 or Ph-WT cells. To quantify these patterns, we classified the regulatory region of the gene housing the viewpoint as ‘near', and the sequences in the *BX-C* outside of this region as ‘far' (Fig. 3a). The average ratios of reads in Ph-WT or Ph-ML cells normalized to those in S2 cells were determined for ‘near' and ‘far' regions. We found that viewpoints in Ph-ML cells have significantly decreased ‘far' interactions within the *BX-C* as compared with Ph-WT cells (Fig. 3c). We also analysed the 4C-seq data using the 4C-seqpipe software developed in the Tanay lab^31^ and plotted interactions in the *BX-C* region. This analysis (Supplementary Fig. 8) is consistent with reduced interactions with sequences far from the viewpoint in Ph-ML cells, and also shows that the overall pattern of contacts is similar in the three cell lines. Thus, in cells with disrupted PcG clustering, long-range (>100 kb) chromatin interactions inside the chromatin topological domains comprising the *BX-C* are impaired. We note that we are interpreting the changes in the 4C-seq data as changes in chromatin contacts. Although the validity of this interpretation is supported by many published studies, a careful comparison of 5C data to FISH experiments (which can measure how close together two sequences are in the nucleus) found that the two methods are not always concordant^33^. Thus, we cannot rule out the possibility that some of the contacts detected in our analysis may not directly reflect physical proximity, which would make the interpretation of differences among cell lines difficult for these contacts.

### Ph SAM mutations do not alter binding to most genomic sites

To test the relationship between chromatin contacts and Ph binding, we first mapped the distribution of Ph-WT and Ph-ML genome wide using chromatin immunoprecipitation followed by sequencing (ChIP-seq; Fig. 3b, Supplementary Fig. 9). Approximately 6,300-binding sites are shared in both cell types, indicating that the global distribution of Ph-WT and Ph-ML are very similar. Careful quantification of our ChIP-seq data indicates that Ph-ML levels are reduced relative to Ph-WT levels at a subset of Ph-binding sites (∼4%), including many in the *BX-C* (Supplementary Fig. 9). To determine the effect of overexpression of Ph on genome wide distribution, we also compared the distribution of Ph-WT to endogenous posterior sex combs (PSC) (another PRC1 subunit) using previously published data (GSE38166)^34^. We find that 93% of Ph-binding sites overlap with PSC, with an additional 485 sites detected in Ph-WT and Ph-ML cells.

### Ph binding and chromatin contacts in the *BX-C*

To test the relationship between Ph binding and chromatin contacts in the *BX-C* region, we measured the distance between each contact and the nearest Ph-binding site (Fig. 3b). We classified contacts in Ph-ML and Ph-WT cells according to whether they are changed by >50% relative to S2 cells. We found that contacts that decrease in Ph-ML cells are closer to Ph-binding sites than contacts that are unchanged (*P*=0.0021, Student's *t*-test; Fig. 3d). Contacts that increase in Ph-WT cells are also closer to Ph-binding sites than contacts that are unchanged (*P*=2.25e-06, Student's *t*-test; Fig. 3d). Thus, SAM-dependent Ph–Ph interactions, which are disrupted in Ph-ML cells and may occur more frequently in Ph-WT cells, may directly mediate these contacts. In Ph-ML cells, reduced binding at Ph sites in the *BX-C* may also contribute to the decrease in chromatin contacts.

### Ph manipulations alter long-range contacts detected by 4C

**Figure 4:**
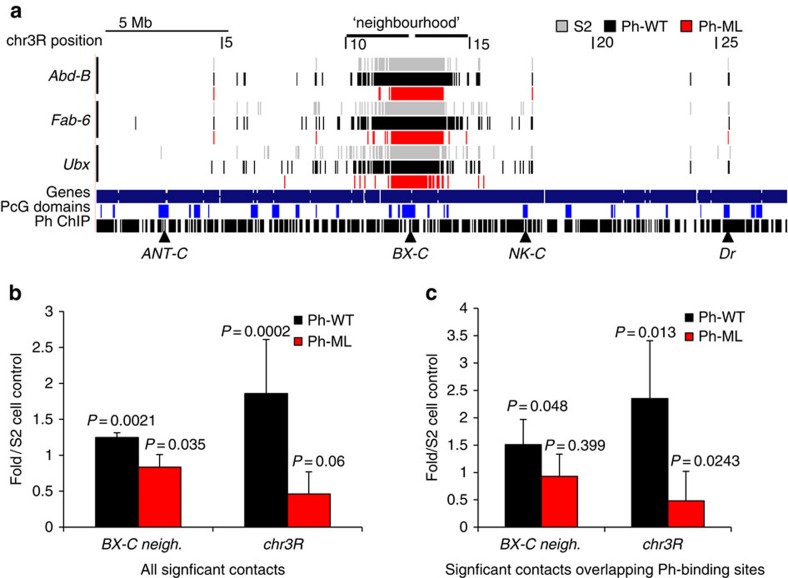
Long-range contacts between the *BX-C* and regions on chromosome 3R. (**a**) Significant contacts identified using *fourSig*^35^ between viewpoints within the *BX-C* and chromosome 3R. See also Supplementary Fig. 10. Triangles mark the *BX-C* and some PcG domains previously identified as interacting with the *BX-C* by 4C analysis in *Drosophila* embryos^15^ and/or in larval brain tissue^14^. Tracks show data from one of two replicates; both replicates are shown in Supplementary Fig. 10a, (**b**) Quantification of long-range contacts within 2 Mb of the borders of the *BX-C* (*BX-C* neighbourhood), or further than 2 Mb away from the *BX-C* on chr3R. Contacts were normalized to those in control S2 cells and averaged for all three viewpoints. (**c**) Quantification of long-range contacts that overlap Ph-binding sites as in **b**. *P* values are for paired, two-tailed Student's *t*-test comparing the number of contacts between Ph-ML and S2 or Ph-WT and S2 for each of two replicates with each of the three viewpoints. For the comparison between Ph-ML and S2 cells for chr3R in **b**, data violated the assumption of normality by Anderson Darling test. Using a 1 Sample Wilcoxon Exact Test, the two-sided *P* value is 0.03. All other data sets passed the assumption of normality by Anderson Darling test.

To analyse contacts between viewpoints inside the *BX-C* and more distal sequences, we used the programme *fourSig* (ref. 35) to identify significant 4C-seq interactions. We found that significant contacts were detected between the viewpoints in the *BX-C* and other regions of chromosome 3R on which it resides (Fig. 4a, Supplementary Fig. 10a). In all, 60–70% of identified 4C contacts overlap at least one Ph-binding site, with the highest percentage found in cells expressing extra Ph-WT (Supplementary Fig. 10b,c). These contacts may reflect Ph–Ph interactions. Contacts made with sites that do not bind Ph may reflect additional molecular mechanisms (such as Ph interactions with other proteins), or limitations in our 4C analysis. Ph-WT-expressing cells form more and Ph-ML cells fewer contacts between the *BX-C* and other sequences on chromosome 3R than S2 cells (Fig. 4b,c, Supplementary Fig. 10d). To determine whether these differences reflect long-distance interactions or predominantly those with sequences close to the *BX-C*, we parsed the contact data into the *BX-C* neighbourhood (2 Mb on either side of the *BX-C*), and more distal sequences on chromosome 3R (>2 Mb from the *BX-C* on either side). The same trends were observed both for the *BX-C* neighbourhood and for distal regions of chromosome 3R, but were more striking for more distal contacts (Fig. 4b,c). We conclude that disrupting PcG protein clustering by interfering with the polymerization activity of Ph SAM decreases chromatin contacts within the *BX-C* and long-range intra-chromosomal contacts, whereas increasing levels of polymerization-competent Ph-WT increases long-range chromatin contacts.

### Molecular simulation of Ph SAM-dependent PcG clustering

To gain mechanistic insight into the relationships among PcG cluster size, cluster number and long-range chromatin interactions, and how these parameters are regulated by Ph SAM, we carried out molecular simulations using the ‘strings and binders' model for chromatin folding^36^. The chromatin backbone is represented as a self-avoiding random walk polymer, which may be bound by Ph molecules. Clusters of Ph may form and grow in two ways: (1) ‘spreading' when multiple molecules may bind and polymerize at the same locus (that is, node on the polymer); (2) ‘bridging' when molecules or clusters bound at a node polymerize with those bound at other, non-adjacent nodes on the chromatin polymer, forming a long-range chromatin contact. Individual molecules may also leave a cluster and return to the solution and molecules at nodes linked by long-range interactions may dissociate (see Methods section for full description of model and parameters). For the initial model, spreading at any locus was limited to 15 copies of Ph.

**Figure 5:**
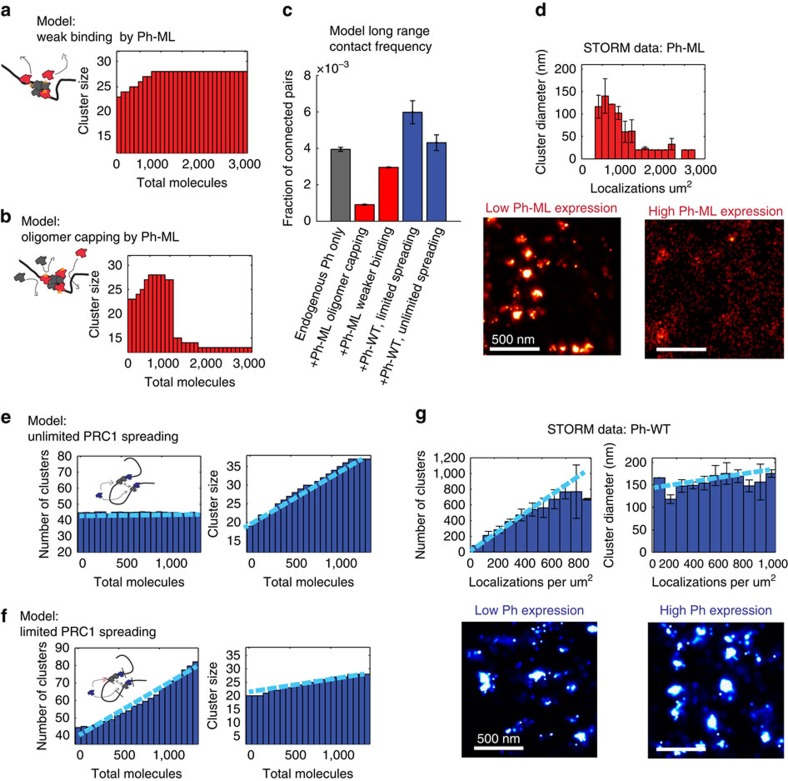
Molecular modelling of Ph SAM-dependent organization of PcG proteins and chromatin topology. (**a**,**b**) Simulations of the effect of Ph-ML concentration on cluster size in either the weak binding (**a**) or oligomer capping (**b**) models. (**c**) Quantification of the frequency of long-range contacts in the baseline model with no added Ph (grey), the models with added Ph-ML assuming weak binding or capping (red) and the models with added WT Ph assuming unlimited or limited chromatin spreading (blue). Bars show the average contact frequency from multiple simulations across the range of concentrations of Ph-ML or Ph-WT used. Error bars represent S.E.M. over repeated simulations. (**d**) Experimental results for total Ph clustering as a function of Ph-ML concentration. Error bars represent S.E.M. Lower panels show STORM images from cells at low- and high-Ph-ML concentration. Grey, Ph; red, Ph-ML. (**e**,**f**) Number of clusters (left graphs) and median cluster diameter (right graphs) as a function of Ph concentration in simulations with unlimited spreading of Ph on chromatin (**e**) or with limited (15) Ph-binding sites per node (**f**). (**g**) Number of clusters and median cluster diameter as a function of Ph-WT concentration from STORM measurements. Right panels are STORM images at low- and high-Ph-WT concentrations. Light blue lines in **e**–**g** are to indicate the data trend but are not data fits. Scale bars, 500 nm.

We used this model to explore two different mechanisms for how mutations in Ph SAM affect PcG clustering and chromatin interactions. In the first case, Ph-ML has a higher *k*_off_ from clusters due to weaker interactions; in the second case, Ph-ML binds as well as wild type, but reduces binding (*k*_on_) of subsequent Ph molecules to the cluster. These mechanisms make different predictions about the relationship between Ph-ML concentration and cluster size, which are captured in simulations (Fig. 5a,b). For the first mechanism, as Ph-ML concentration increases, cluster size increases slightly and then remains constant once Ph-ML molecules start to compete with endogenous molecules to join nascent clusters, and the increase in binding rate is balanced by the reduction in cluster stability (Fig. 5a). For the second mechanism, cluster-size first increases slightly as the new Ph-ML molecules join existing clusters and then sharply decreases when there is sufficient Ph-ML to ‘cap' all endogenous clusters and prevent them from either spreading or bridging (Fig. 5b). Reduced bridging interactions decreases cluster size (Fig. 5b). We calculated the frequency of interactions among Ph bound sites (nodes on the polymer) in each simulation and find that these bridging contacts are reduced more by Ph-ML in the oligomer-capping model than the weak binding model (Fig. 5c).

To test whether the effect of Ph-ML on PcG clusters is consistent with either of these models, we took advantage of the cell-to-cell variation in expression of Ph-ML in our cell lines (this variation is due to the non-clonal nature of the lines and the stochastic response of the copper-inducible promoter that drives Ph-ML expression). We computed Ph-ML concentration as the number of individual molecular localizations counted by STORM per square micron, and observe a more than fivefold expression range. We note that because each dye molecule could switch on multiple times and give multiple localizations and because the labelling efficiency is not necessarily 100%, these localization counts do not represent absolute concentrations but can be used for relative comparisons. The median cluster diameter was then plotted against this relative Ph-ML concentration measure (Fig. 5d). This pattern is very similar to the capping model simulation, and distinct from the weak-binding simulation (compare 5d with 5a and 5b), suggesting that the effects of Ph-ML on PcG clustering and long-range chromatin interactions are consistent with the oligomer-capping mechanism.

To analyse the effect of increasing Ph levels (as in Ph-WT cells), we again considered two mechanisms. In the first, we removed the constraint on Ph spreading (that is, the number of molecules that can load at a single node) and in the second we retained this constraint (such that the nodes available for binding will be saturated at high-Ph expression; see schematics in Fig. 5e,f respectively). In simulations of the first model, increasing Ph concentration causes existing clusters to grow primarily by spreading, leading to an increase in average cluster size (Fig. 5e) without increasing the frequency of bridging interactions (Fig. 5c), both inconsistent with our experimental observations (Fig. 5g). In simulations of the second model, as Ph levels increase, the number of clusters rises rapidly as high-affinity nodes are saturated and new molecules bind low-affinity nodes (Fig. 5f). The increase in cluster number increases bridging interactions (because the clusters are more likely to encounter each other), and thus increases long-range chromatin contacts (Fig. 5c). Because new bridges primarily involve nodes unoccupied at lower Ph concentration, they only modestly affect cluster size (Fig. 5f, right graph), qualitatively consistent with our empirical observations of the relation between total Ph concentration and cluster-size distribution (Fig. 5g).

The qualitative differences in clustering and three-dimensional interactions observed in our simulations are observed for a broad range of parameters, provided that the relative differences in affinity are maintained. The results do not depend on the precise values used in the simulations shown here and listed in Supplementary Table 3. The parameter space of these transitions between clustered/condensed and unclustered/open states has been explored carefully in previous theoretical work, and our results fall within the scenarios predicted by polymer thermodynamics^36^,^37^,^38^,^39^.

### Ph levels and PcG clustering affect gene expression

**Figure 6:**
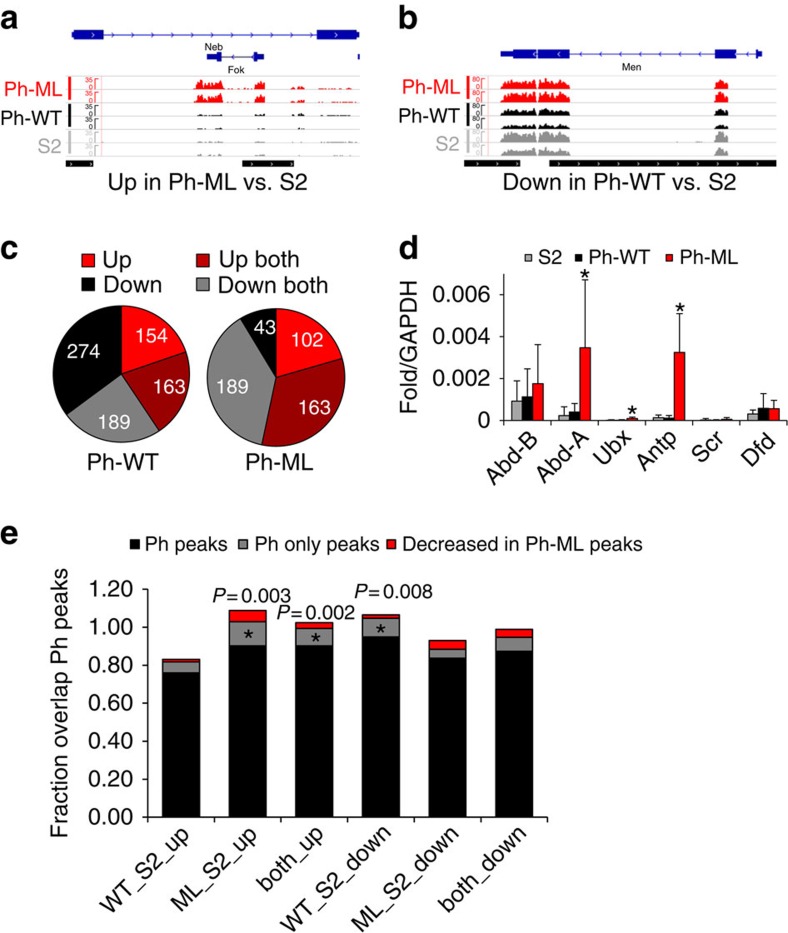
Gene expression changes induced by increased wild-type or mutant Ph. (**a**,**b**) Sample traces from RNA-seq data of genes that are derepressed in Ph-ML cells (**a**), or repressed in Ph-WT cells (**b**). Normalized traces of both replicates of each of the three data sets (S2, Ph-WT, Ph-ML) are shown. Bottom track indicates Ph-binding sites (black). (**c**) Summary of significantly changed genes in Ph-WT (left) or Ph-ML (right) cells compared with S2 cells as determined by RNA-seq analysis. Genes were analysed at the level of transcription start sites (TSS). Significant TSSs were identified using Cuffdiff and further filtered for and fdr corrected *P* value of <0.01, an FPKM of at least five (in one of the two data sets), and a fold-change of at least two. See also Supplementary Figs 12 and 13. (**d**) PCR with reverse transcription analysis of *Hox* gene expression. Asterisks indicate differences with *P*<0.05 by Student's *t*-test. Bars are the average of at least three experiments and error bars show s.d. Note that three primer sets were used to analyse each gene with similar results except that Ubx is only significantly elevated in Ph-ML cells with the primers shown. (**e**) Summary of the overlap between regions with altered gene expression and Ph-binding sites identified by ChIP-seq. Gene categories are the same as in **c**. Black bars show overlap with all Ph peaks, grey bars show overlap with peaks identified as Ph-binding sites in Ph-WT cells but not as PSC-binding sites in S2 cells, and red bars show overlap with peaks that have decreased binding of Ph-ML relative to Ph-WT. The *P* values (permutation test) for overlaps of all Ph peaks with the different gene sets (black bars) are all <0.005. For grey and red bars where *P* values are not stated, *P*>0.05 (permutation test); stated *P* values are for bars indicated with asterisks.

To determine the functional consequences of increasing or decreasing PcG protein clusters, we compared gene expression in normal S2 cells, and those expressing Ph-WT or Ph-ML (in addition to endogenous Ph) by RNA-seq. We carried out two biological replicates and mapped the resulting reads to the *Drosophila* genome using TopHat. We used Cufflinks to identify significant differences between Ph-ML, Ph-WT and S2 cells. Global correlations between different pairs of samples indicate that transcriptomes of Ph-ML and Ph-WT are more similar to each other than to S2 cells (Supplementary Fig. 11a). Out of 925 transcripts with altered expression, 352 were similarly regulated in both Ph-WT and Ph-ML cells (Fig. 6c, Supplementary Fig. 11b–e). Regulation of these genes therefore depends on Ph but likely not on the polymerization capacity of Ph SAM. It has been reported that decreasing and increasing Ph levels can have similar effects on gene expression and tumour phenotypes in imaginal discs^23^,^40^,^41^,^42^, which may be due in part to interfering with stoichiometries of PcG complexes (as PRC1 components assemble into multiple complexes, only some of which contain Ph (refs 43, 44)).

Genes that are uniquely up- or downregulated in either Ph-ML or Ph-WT were also identified. These changes in gene expression likely depend on Ph SAM polymerization capacity, and may thus reflect effects of PcG protein clustering. Of the uniquely regulated genes most were downregulated in Ph-WT versus S2 (64%), whereas most were upregulated in Ph-ML versus S2 (70%) (Fig. 6a,b). These effects are consistent with the expected repressor function of Ph-WT and dominant negative interference with repression by Ph-ML. We also observe a substantial number of genes upregulated in Ph-WT cells or downregulated in Ph-ML cells. These effects may be downstream consequences of (respectively) repressing or de-repressing negative regulators of transcription). It is also possible that Ph SAM polymerization capacity is important for activation of some genes. The best studied targets of Ph regulation are the developmentally important *Hox* genes. In our RNA-seq analysis, *Hox* gene sequences were not sufficiently represented to be included in the analysis. We therefore used PCR with reverse transcription to analyse expression of *Hox* genes in the *BX-C* and *ANT-C*. We find that *abd-A* and *Antp* are derepressed in Ph-ML cells, but not Ph-WT cells, consistent with previous observations of Ph SAM mutants (Fig. 6d). We conclude that Ph SAM polymerization capacity, which is important for PcG protein clustering, is also important for the repressor function of Ph at some genes. We cannot rule out the possibility that some effects of Ph-ML reflect disrupted interactions with SCM, which is also essential for PcG repression.

To investigate whether changes in gene expression are likely to be direct effects of Ph binding, we queried the overlap between ChIP-seq peaks and differentially regulated genes (Fig. 6e). For all sets of differentially expressed genes, significant overlap with Ph peaks was observed (*P*<0.005, permutation test). We also identified a set of ectopic peaks in Ph-WT and Ph-ML cells (485 peaks, noted above) that were not identified as binding sites for PSC. Although some of these sites may reflect technical limitations of ChIP-seq, some likely represent *bona fide* sites induced by overexpression. We therefore asked if these peaks overlap differentially expressed genes. Overlap was distinct from random (*P*<0.005, permutation test) for genes upregulated in Ph-ML versus S2, upregulated in both Ph-ML and Ph-WT versus S2, and downregulated in Ph-WT versus S2 (Fig. 6e). To determine what role loss of Ph binding at some sites might have in gene regulation, we also compared overlap between peaks with decreased binding of Ph-ML to differentially expressed genes, but this overlap was not significant for any of our comparisons.

Our results are consistent with the findings of Gambetta & Mueller^24^, who showed that Ph lacking the SAM cannot replace *ph* function during *Drosophila* development. Ph with a mutation in the EH polymerization interface of the SAM is able to partially rescue *ph* function. Taken together, it seems that Ph SAM is critical for gene regulation by the PcG, both through mechanisms that depend on its polymerization capacity and mechanisms that do not (but may still involve protein–protein interactions).

## Discussion

Our major finding is that PcG proteins form hundreds of nano-clusters, whose characteristics are determined by the polymerization activity of Ph SAM. Using STORM imaging expanded the number and sizes of PcG protein clusters observed, allowing us to characterize these changes in greater detail than possible previously. By manipulating PcG protein clusters and monitoring the effect on chromatin topology, we find that PcG clustering facilitates long-range chromatin interactions as interactions are decreased when clustering is disrupted and increased when clustering is enhanced. These changes result in corresponding changes in gene expression, including de-repression of some well-known PcG target genes such as *Antp* and *abd-A* when clustering is disrupted and ectopic repression of other loci when cluster counts are increased. Our observations are consistent with a model in which Ph SAM mediated clustering of PcG proteins occurs at hundreds of sites in the genome and facilitates both folding of PcG-bound sites into domains like the *BX-C* (Fig. 3) and long-range interactions among PcG-bound sites (Fig. 4). These architectural effects may contribute to the efficiency of PcG-mediated gene regulation. Molecular simulations provide mechanistic details into cluster regulation and how it is linked to long-range chromatin contacts. These simulations suggest how recruitment of PcG proteins to genomic loci with different affinities and PcG binding capacities regulates the size and number of clusters that form, and thus the extent of long-range chromatin interactions. The clustering of PcG proteins has been hypothesized to mediate interactions among PcG-bound genes^2^,^15^,^25^,^45^ but it has been difficult to reconcile this idea with the relatively low frequencies of long-range interactions detected among large PcG domains in genomic and microscopy based studies^11^,^15^,^46^. We suggest that PcG protein clustering does indeed facilitate long-range interactions, but that these interactions are occurring among hundreds of cluster-bound sites rather than a few PcG bodies. Very recently, a PRC1-dependent network of physical interactions among *Hox* gene promoters and promoters of other PcG-bound genes encoding developmental regulators was described in mouse embryonic stem cells^47^. It is not yet known if this global organization depends on the polymerization activity of Ph SAM, but that would be consistent with our model. Finally, given that many nuclear proteins have been observed to form clusters, it is possible that not-yet-described nanoscale organization of these proteins also contributes to orchestrating diverse nuclear processes.

## Methods

### Cells lines and protein purifications

*Drosophila* S2 cells were obtained from Expression Systems and cultured on plates or in shaker flasks at 27 °C in ESF921 media (Expression Systems). Constructs for expressing epitope tagged mutant and Ph-WT were generated by cloning Ph into a pMT vector modified to encode tandem 2XFLAG and biotin ligase recognition peptide (BLRP) sequences (note that this construct was described previously^34^,^48^. *Drosophila* has highly similar tandem copies of the *ph* gene, *ph-proximal* and *ph-distal*. *ph-proximal* was used in all of this work. Ph SAM mutations were generated by site-directed mutagenesis. All Ph-coding sequence was verified by sequencing. Stable cell lines were generated by transfecting cells with plasmids encoding BLRP-2XFLAG-Ph (Ph-WT) or BLRP-2XFLAG-Ph-L1547/H1552R (Ph-ML) and a plasmid encoding the *E. coli* Biotin ligase (BirA) and the puromycin resistance gene (Supplementary Fig. 2). Both Ph variants and BirA are controlled by the metallothionein promoter. For some experiments (Supplementary Fig. 10), cell lines were made with a plasmid-expressing green fluorescent protein constitutively along with the Ph and BirA plasmids. Green fluorescent protein-expressing cells were isolated by FACS to increase the fraction of Ph-expressing cells, and propagated as stable lines.

Ph-WT, Ph-EH or Ph-ML expression was induced by treatment for 3 days with 0.5 mM copper sulphate. To purify Ph-associated complexes, nuclear extracts were prepared from 1 l of induced cells. Nuclear extracts were prepared essentially as described^49^. Detailed protocols are available on request. For purification of PcG complexes, nuclear extracts were incubated with anti-FLAG beads overnight at 4 °C on a rotator, washed 2 × with BC300N (20 mM hepes, 20% glycerol, 0.2 mM EDTA, 0.05% NP40, 300 mM KCl, pH 7.9), 2 × with BC600N and 2 × with BC1200N, and once again with BC600N and BC300N. PcG complexes were eluted in BC300N containing 0.4 mg ml^−1^ of 2X FLAG peptide. Protease inhibitors (0.2 mM PMSF, 10 μg ml^−1^ leupeptin, 10 μg ml^−1^ aprotinin, 2 μg ml^−1^ pepstatin, 16 μg benzamidine, 10 μg ml^−1^ phenanthroline, 50 μg ml^−1^ N-a-tosyl-L-lysine chloromethyl ketone hydrochloride (TLCK)) and dithiothrietol (DTT) (0.5 mM) were added to all buffers and extracts, and all procedures were carried out at 4 °C. For tandem anti-FLAG and streptavidin purification, FLAG elutions were pooled and incubated overnight with M280 streptavidin-coated Dynabeads. Beads were captured and washed five times with BC300N, once with BC50N and boiled in sample buffer to release purified proteins. Expression of PRC1 in Sf9 cells and subsequent purification were as described^48^ except that nuclei were purified through a sucrose cushion before extraction.

### Density gradient centrifugation

A measure of 4 ml 10–40% glycerol gradients were prepared manually by layering 200μl of solutions with decreasing amounts of glycerol in BC300N. Anti-FLAG (100 μl) purified PcG complexes were mixed with 100 μl of BC300N (containing no glycerol) to make the final glycerol concentration to 10%. A measure of 200 μl of this mix were loaded on the gradients, and centrifuged for 6 h at 103,400*g* in an SW55Ti rotor at 4 °C in a Beckman ultracentrifuge. Fractions (200 μl) were collected and analysed by western blotting. Western blots were quantified using Image J software. The gradient input was included on each gel to serve as a standard for quantification of fractions from the same gradient across gels. Supplementary Fig. 12 shows examples of full blots used for quantification. Primary antibodies for analysis of gradients were anti-Ph (kind gift of J. Mueller), anti-PSC (generated in our lab), anti-dRING (kind gift of R. Jones) and anti-Pc (kind gift of J. Mueller).

### Immunofluorescence

*Drosophila* S2 cells and S2 cell lines expressing Ph-WT or Ph-ML were grown on Concanavalin A-coated coverslips for 1.5 h. Cells were washed with phosphate-buffered saline (PBS), fixed with 4% formaldehyde (in PBS) for 10 min and washed twice with PBS. Freshly prepared sodium borohydride (1 mg ml^−1^) solution was added to cells for 7 min, and cells were again washed twice with PBS. Cells were incubated in permeabilization buffer (1 × PBS, 0.02% Tween-20 and 0.1% Triton X-100) for 15 min. Two washes of PBST (1 × PBS, 0.02% Tween-20) were carried out before blocking cells 30 min in PBST containing 2% BSA. Primary antibodies diluted 1:200 in PBST+2% BSA were added to coverslips and incubated overnight at 4 °C. After washing three times with PSBT* (1 × PBS, 0.1% Tween), cells were briefly blocked again for 5 min in PBST*containing 2% BSA. Appropriate secondary antibodies (diluted 1:200 in PBST*+2% BSA) were added to coverslips for 1 h. Coverslips were washed three times with PBST* and mounted for imaging. All procedures were carried out at room temperature unless otherwise indicated. Three primary antibodies were used, anti-Ph, anti-Pc (kind gifts of J. Mueller) and M2 anti-FLAG (Sigma F1804). Secondary antibodies (donkey anti-rabbit, R&D systems D-301-C-ABS2, donkey anti-mouse, R&D systems D-201-C-ABS2, and donkey anti-mouse Jackson Immunoresearch, 715-005-150) were conjugated to NHS-ester reactive Alexa-Fluor 405, Alexa-Fluor 647, Alexa-Fluor 750 or Cy7 dyes. An average labelling ratio of 2 Alexa-Fluor 405 dyes and 1–2 647 dyes or 2–4 Alexa-Fluor 750 or Cy7 dyes per antibody was confirmed by absorption spectroscopy.

### STORM imaging

Cells were imaged on a customized Olympus IX-71 inverted microscope configured for oblique incidence excitation. Microscope configuration for sequential dual colour 750/647 imaging using a Quadview set-up was performed as previous described^50^. This configuration requires no moving parts (for example, filterwheels) to switch between chromatic channels, thus reducing alignment error between channels. This set-up has a resolution of 20–25 nm for the Alexa 647 channel and 25–35 nm for the Alexa 750 channel^20^,^21^. Images of fiducial beads (715/750 FluoSpheres, Life Technologies) visible in both the 750 and 647 channel were acquired prior data acquisition and used later to correct for chromatic aberrations (see below). Fiducial beads (200 nm 540/560 FluoSpheres, Life Technologies), illuminated with a 561 laser were simultaneously tracked during image acquisition. Movies of Alexa750 and Cy7 dyes were taken for 20,000–30,000 frames at 60 Hz. Movies of Alexa647-labelled samples were taken for 60,000–100,000 frames at 60 Hz. 405 activation laser intensity was ramped during a calibration movie for each data set (0.1–30 W cm^−2^) to maintain an approximately constant rate of photo-switching events per frame. The same activation laser ramp and number of frames was then used for all samples in a data set. Imaging buffer was used as previously described for single-colour imaging with Alexa-Fluor647 (ref. 51) using *β*ME as a thiol. Dual-colour imaging was performed with 0.5% (v/v) *β*ME instead of 1% and with an addition of 1% (v/v) cyclooctatetraene (2M in DMSO), to increase photon count per switching cycle^52^.

### Image analysis

Fluorophores were localized using a previously described multi-fitting algorithm^53^. Bead trajectories (imaged at 60 Hz) from fiducial tracking during each image were smoothed with 200 frame averaging window to improve localization accuracy and used to correct stage drift in the STORM images. For multi-colour data, images of fields of 100 nm 715/755 FluoSpheres and 200 nm 540/560 FluoSpheres acquired before and after STORM images were used to compute polynomial chromatic corrections. Typical alignment error for these maps was ∼10 nm. These maps were then applied to the multi-colour STORM data after drift correction for aligning the two colour channels.

Clusters were determined as follows: the positions of all detected molecules in the cell were discretized into small bins (typically ∼15 × 15 nm, see below). Any connected set of non-empty bins surrounded by empty bins was considered to be a cluster (including isolated, single bins). Non-empty bins that are adjacent or diagonal to one another were considered connected. Bin size was adjusted for variation in the molecule count density following the equation: bin size=15 nm × (average localizations per cell/localizations in this cell)^1/2^. The range of sizes was further restricted to be between 10 and 30 nm. The resulting bin sizes were 15±2.5 nm (mean±std). This adjustment improved the agreement between the automated cluster calls and the visual impression of clustering across the data set.

Colocalization analysis was performed by binning the localizations detected in each channel into 15 × 15 nm bins. The resulting bins were clustered in each channel separately, based on being surrounded by non-empty bins as described above. Clusters smaller than our 30 nm resolution limit (single bins) were excluded from this analysis. Clusters which overlapped (contained at least one bin in which localizations from both channels were detected) were considered to be colocalized. We then report the fraction of localizations which were assigned to clusters considered to be colocalized relative to the total number of localizations from all clusters. We also refer to this ratio as the degree of overlap between the channels.

### ChIP-seq

ChIP was carried out essentially as described^34^. *Drosophila* S2 cell lines-expressing Ph-WT or Ph-ML were induced for 3 days, fixed with 1% formaldehyde for 10 min at RT and quenched with 1 M glycine, pH 7.9. Cells were washed with 1 × PBS, wash buffer I (10 mM hepes, pH 7.6, 10 mM EDTA, 0.5 mM EGTA, 0.25% Triton-X-100), and wash buffer II (10 mM hepes, pH 7.6, 200 mM NaCl, 1 mM EDTA, 0.5 mM EGTA, 0.01% Triton-X-100). Cells were centrifuged and resuspended in sonication buffer (50 mM hepes, pH 7.5, 500 mM NaCl, 1 mM EDTA, 1% Triton X-100, 0.1% sodium deoxycholate, 0.1% SDS) to a cell concentration of 20 × 10^6^ cells ml^−1^. Cells (1 ml) was sonicated with 4 × 30 s pulses with 30 s between pulses using a Sonics Vibracell sonicator at 40% power. Following sonication, samples were centrifuged for 5 min at 14,000 r.p.m. in a microcentrifuge at 4 °C. The supernatant was used for ChIP. Sonicated chromatin (400 μl) were mixed with 600 μl of ChIP buffer to make buffer concentration to 1 × ChIP buffer (15 mM Tris, pH 8, 150 mM NaCl, 1 mM EDTA, 1% Triton X-100 and 0.01% SDS). The mixture was incubated with beads from 144 μl of M280 streptavidin magnetic beads (Invitrogen) slurry, which were blocked with 0.2 mg ml^−1^ salmon sperm DNA, and washed with ChIP buffer, overnight at 4 °C on a rotator. Beads were washed 3 × with 1 ml of ChIP buffer, resuspended in 300 μl elution buffer (0.5 M NaCl, 1% SDS) and incubated overnight at 65 °C. Input samples were also mixed with appropriate volume of buffer to make final buffer conditions identical to 1 × elution buffer and all samples were processed similarly from here onwards. A measure of 1 μl of RNase A (30 μg) was added to each sample and samples were incubated for 30 min at 37 °C. Samples were treated with proteinase K (20 μg) for 1 h at 50 °C and DNA was purified using the Nucleospin Extract II kit (Macherey-Nagel). Sequencing libraries were generated as described previously^54^. In brief, ends of the immunoprecipitated DNA were repaired, followed by A-tailing, ligating to universal adaptors and amplification for 10 cycles with indexed primers. Excess adaptors were removed by purification with Agencourt AMPureXP beads (Beckman-Coulter). Fragment size was checked by Bioanalyzer using a high-sensitivity DNA chip and an average size distribution of 300–400 bp was observed.

### ChIP

ChIP experiments to validate binding patterns of Ph-WT and Ph-ML proteins were carried out essentially as described above. ChIP with antibodies against PSC (our lab), Ph (kind gift of J. Mueller), H3 (Abcam ab1791, ChIP grade) and H3K27me3 (Abcam, ab6002, ChIP grade) were carried out essentially as in ref. 34 with the following exceptions. Chromatin (100 μl, corresponding to 2e^6^ cells) was diluted to 1X ChIP dilution buffer, and pre-blocked with protein G sepharose (gammabind G, GE Healthcare) that had been incubated with 1% BSA-0.2 mg ml^−1^ yeast tRNA. Antibodies (4 μg per ChIP) pre-bound to 16 μl of Protein G Dynabeads (Invitrogen) were added to chromatin and samples were incubated overnight at 4 °C with rotation. Washes were carried out for 10 min each at 4 °C with rotation as follows^55^: 1X RIPA (10 mM Tris, pH 8.0, 140 mM NaCl, 1 mM EDTA pH 8.0, 1% Triton X-100, 0.1% sodium deoxycholate, 0.1% SDS), 3X RIPA-500 mM NaCl, 1X LiCl wash (10 mM Tris, pH 8.0, 0.25 M LCl, 1 mM EDTA, 1% NP40 and 1% sodium deoxycholate), 2X TE. Samples were eluted by incubating with 0.5 M NaCl/1% SDS/0.1 M NaHCO_3_ at 65 °C twice for 15 min each. To reverse cross-links, ChIP elutions and input samples were incubated for 15 min at 95 °C. Samples were treated with RNaseA and Proteinase K, and purified as described above. Purified DNA was quantified by real-time PCR with SYBR green on a ViiA7 instrument. PCR was run in ‘standard curve' mode with genomic DNA from S2 cells as the standard. Primer sequences were mostly previously described^34^; additional sequences are in Supplementary Table 1.

### 4C-seq

After 3 days of induction, *Drosophila* S2 cells and S2 cells expressing Ph-WT or Ph-ML were cross-linked with formaldehyde as described above. Nuclei were prepared by incubating fixed cells in Buffer A (20 mM hepes pH 7.5, 1.5 mM MgCl_2_, 10 mM KCl and 0.1% NP40) for 12 min on ice, douncing 60 times and centrifuging at 150 g for 1 min at 4 °C. The supernatant was centrifuged at 1,000 g for 10 min, and pelleted nuclei were washed with PBST. 4C-seq was carried out as described elsewhere^31^. In brief, Nuclei were resuspended in 180 μl of water and 20 μl of 10 × NlaIII buffer. Nuclei were digested with NlaIII for 20 h at 37 °C in a thermo-shaker set at 1,000 r.p.m. NlaIII was inactivated by incubating samples at 65 °C for 10 min. Digested DNA was ligated with T4 DNA ligase overnight at 16 °C. Ligation mixture was treated with Proteinase K, cross links were reversed by overnight incubation at 65 °C, and RNase A treatment was performed for 30 min at 37 °C. Phenol–chloroform extracted and ethanol precipitated DNA was digested with DpnII for 7 h at 37 °C in a thermo-shaker set at 1000, rpm. DpnII was inactivated by incubating samples at 65 °C for 15 min and DNA was ligated with T4 DNA ligase overnight at 16 °C. DNA was purified by phenol–chloroform extraction and ethanol precipitation and resuspended in 30 μl of water. DNA (20 ng) per 50 μl of PCR reaction mixture was amplified for 30 cycles by expanded long-range DNA polymerase (Roche) using indexed viewpoint primers. Base pair (100) single-end reads were sequenced at the Genomics Platform, University of Genève. Note that the sequenced primer corresponds to the secondary (DpnII) junction rather than the primary (NlaIII) one. Primers used for 4C-seq are in Supplementary Table 2.

### HTS-pipeline analysis of 4C-seq inside the *BX-C*

4C-seq reads were de-multiplexed and aligned to the *Drosophila* genome using the HTSstation pipeline (http:// htsstation.vital-it.ch/)^32^. Reads were normalized over a region encompassing the *BX-C* (chr3R:12367359:12885749) and raw interaction frequencies smoothened using a running mean of three fragments as described previously^56^. The region 5 kb up- and downstream from the viewpoint sequence was excluded from the analysis. These interaction frequencies were averaged over the two experiments to create the tracks displayed in Supplementary Fig. 8. To create Fig. 3a, Ph-WT and Ph-ML normalized frequencies were divided by those from S2 cells; 1 was subtracted from the ratios so that increased frequencies are positive and decreased ones negative. To analyse the pattern of ‘near' versus ‘far' contacts for the *Abd-B* and *Fab-6* viewpoints (both of which fall within the regulatory region of *Abd-B*), the ‘near' region was defined by the boundary between the *iab3* and *iab4* elements (chr3R:12681222), which regulate *abd-A* and *Abd-B*, respectively. To analyse ‘near' versus ‘far' contacts for the *Ubx* viewpoint, the proximal region was defined by the start of the *bxd* non-coding transcript (chr3R:12598911). Note that this analysis was carried out using several different boundary demarcations for ‘near' versus ‘far' (for example, *iab3/4* for all three viewpoints, the *bxd/pbx-iab-2* boundary for *Ubx* or defining ‘near' as the 70 kb on one side of the viewpoint) with nearly identical results. For this analysis, the boundaries of the *BX-C* used were chr3R:12480479-12821577.

### 4C-seqpipe analysis of 4C-seq data

4C-seqpipe analysis was carried out as described^31^. Because our primers were designed to read second enzyme (DpnII) junction rather than the primary (NlaIII) one, DpnII was designated as the first cutter and NlaIII as the second. Contacts were calculated from chr3R:12100000–13100000.

### *fourSig* analysis of 4C-seq data

To prepare 4C-seq data for analysis, barcode sequences and the ligation junction were removed using fastxtools (http://hannonlab.cshl.edu/fastx_toolkit). Sequences were further trimmed to 35 base pairs to allow mapping of even very short-ligated fragments and aligned to the *Drosophila* genome (dm3) using Bowtie2 (ref. 57). Aligned reads were used as the input for *fourSig* to create tables of reads mapped to restriction fragments. All analysis was carried out using the ‘mappability' function of *fourSig*.

For details on *fourSig*, please see ref. 35 and the accompanying tutorial http://starmer.med.unc.edu/~jstarmer/fourSig/TUTORIAL.html. The *fourSig* programme identifies significant contacts by determining a threshold number of reads for a given window size that is higher than the background. For all of our analyses, the FDR for the #reads per window that is significant was set to 0.001, and 1,000 random shuffling steps were used to generate the background threshold. The ‘fdr.prob' parameter which is fraction of iterations that exceed the FDR was set to 0.01. *FourSig* also prioritizes interactions based on how ‘broad' the interaction is. Thus, the most stringent interactions remain significant if the #reads/window remains significant after removing the fragment with the highest number of reads. We used only the most stringent contacts for *BX-C* for analysis. *FourSig* generates tables of merged contiguous windows where significant contacts are observed. These tables were loaded directly into the UCSC genome browser for visualization and have been uploaded to GEO. The contacts identified in different experiments are different lengths. Thus, to compare contacts among different cell types, we used BEDtools^58^ to convert the merged windows to the number of restriction fragments. Graphs (Fig. 4b, Supplementary Fig. 4c) show the average number of fragments in 4C contacts in Ph-WT or Ph-ML cells normalized to fragments in contacts from S2 cells.

For the analysis of chromosome 3R, we used a 25-fragment sliding window. Approximately 9 kb of sequence spanning the viewpoint was excluded from the analysis. A more limited set of tests with smaller or larger window sizes also showed the same trends in the data. To analyse the overlap between contacts identified by *fourSig* and Ph ChIP peaks, we used the BEDtools intersect function.

### ChIP-seq Analysis

Sequencing was performed using Illumina Hi-seq 2000. Two biological replicates of ∼45–50 million (per sample), 50 bp, single-end reads were aligned against the dm3 genome using the BWA aligner with an alignment rate of at least 88% (ref. 59). The average fragment length was ∼300–400 bp. We filtered the alignments for uniquely mapped reads and removed PCR duplicates, resulting in 16–28 million reads per sample. We calculated input-subtracted read densities using SPP and calculated SPP broad peaks at fdr<0.005 to predict Ph-binding sites^60^. The final set of Ph peaks was defined as the overlapped peak regions in Ph-ML replicate samples. We calculated the normalized coverage over the peaks regions in both Ph-WT and Ph-ML samples and averaged over replicates. Read densities over peaks were characterized as being similar in Ph-WT and Ph-ML or deviating from the fitted regression line using a cutoff of 16 for the residual values. We identified 258 sites that had decreased binding of Ph-ML relative to Ph-WT and 17 sites that had increased binding.

### Analysis of 4C contacts and Ph-binding sites

To analyse the relationship between genomic loci that reduced interaction frequency with the *Fab6/Ubx/Abd-B* viewpoints and the genomic position of Ph-binding sites, we first determined for 200 bp windows across the *BX-C* locus if the interaction rate was reduced by >50% relative to wild type using the average change across the two biological replicate data sets. We then measured the distance from these points to the centre of the nearest Ph peak using a K-nearest neighbours search (Matlab 2014a) and compared this average distance to the average distance for points that changed by <50% relative to wild type. The Ph peak regions were identified as described above. All called peaks were used as candidate nearest peaks without consideration of peak height or whether peak height changed in Ph-ML. An identical approach was used to handle loci with increased interaction frequency in Ph-WT cells.

### RNA-seq

After three days of induction with 0.5 mM CuSO_4_ *Drosophila* S2 cells, S2 cells or S2 cells expressing either Ph-WT or Ph-ML were harvested, and total RNA was extracted using Qaigen RNA easy kit. RNA (5 μg) from each sample were treated with Ribozero gold kit (Epicenter) and Trueseq kit (Illumina) to deplete ribosomal RNA and generate double-stranded DNA fragments (∼200 bp), respectively. Sequencing libraries were generated as for ChIP-seq. Libraries were sequenced on Illumina Hi-seq 2000 for 50 bp single-end reads. Reads were mapped to the Drosophila genome (release 5) using TopHat (91–92% alignment). We then used Cufflinks with Cuffmerge to assemble the transcripts, and Cuffnorm and Cuffdiff to normalize and compare the transcriptomes^61^. To generate the final lists of differentially regulated genes, we filtered genes for a corrected *P* value of <0.01, fragments per kilobase of transcript per million mapped reads (FPKM) of at least five in one of the two data sets being compared and a fold-change of at least two. The overall trends in the data are identical if less stringent filters are used. We present analysis at the level of transcription start sites rather than genes. The reason for this is that when using gene-level analysis, some loci contain multiple genes (because these transcripts overlap), while transcription start sites map to single transcripts. All of the analysis presented was carried out at the gene level as well, and the results are very similar.

To analyse the overlap between ChIP-seq peaks and differentially regulated genes, we divided genes into classes of ‘up in both Ph-ML and Ph-WT versus S2', ‘down in both' or up/down in only Ph-ML or Ph-WT. We used regioneR (Bioconductor)^62^ to compare the observed overlap to overlap when ChIP-seq peaks are randomized (using ‘permTest' with ‘randomizeRegions'), or to compare overlaps of genes randomly selected from all *Drosophila* genes to each gene set (using ‘permTest' with ‘resampleRegions' and 1,000 permutations). The latter analysis generated the *P* values shown in Fig. 6d, although the results of the two are very similar.

### PCR with reverse transcription

Total RNA was isolated from S2, Ph-WT, or Ph-ML cells that had been induced with 0.5 mM copper sulphate for 3 days, or frozen 0–12 h *Drosophila* embryos using Trizol (Invitrogen) according to the manufacturer's protocol. RNA (10 μg) were treated with DNaseI (NEB) for 15 min at 37 °C, purified by phenol–chloroform extraction and ethanol precipitated. The VILO kit (Invitrogen) was used for cDNA synthesis using 1.4 μg of DNAseI treated total RNA. cDNA was diluted 1:20 or 1:10 and used for real-time PCR with SYBR green on a ViiA7 instrument in ‘standard curve' mode with *Drosophila* embryo cDNA as the standard. Three primer sets were tested for each gene with essentially the same results; only one is shown in Fig. 6d. Primer sequences are available on request.

### Molecular simulations

The molecular simulations were conducted using a modification of the ‘strings and binders' model^36^, and were implemented in Matlab 2014a. The simulation was initialized with a free random walk polymer containing 400 nodes, *N*_endog._ freely diffusing endogenous Ph molecules and *N*_exog._ freely diffusing exogenous Ph or Ph-ML molecules. To improve efficiency of the polymer simulations from the Barbieri *et al*. implementation, a self-avoiding random walk behaviour was simulated using the ‘pivot algorithm'^63^,^64^. When pivot moves were rejected new moves were proposed from the bond-fluctuation method^37^,^65^. Self-avoiding moves that required breaking one or more bonds between binders were accepted following a Metropolis Algorithm governed by the bond energy, *E*_bond_, of binder–binder interactions^36^, which in our case is also proportional to cluster size, as a cluster with *n* molecules may bind up to *n* members of a distant cluster which it contacts. After each move of the polymer, binders were allowed to bind and unbind to each node with probabilities determined by their mass-action kinetic parameters (*k*_a_^endog.^, *k*_d_^endog.^, *k*_a_^exog.^, *k*_d_^exog.^) and the affinity of the node, *A*_node_. The affinity of the node was added to the model to capture the observation that not all genomic locations are equally strong-binding sites for PRC1 and that molecules are likely to accumulate first at the strong-binding sites. Different affinities for each node were selected as a random constant between 0 and 1, which is multiplied by the mass-action-binding probability. Multiple binder molecules were allowed to bind the same node up to a saturation level, *N*_max_. This effect was meant to capture spreading around a local-binding region/PRE, and *N*_max_ asserts a maximal distance of spreading. Each bound molecule at a node presents an independent surface for additional molecules to bind. Saturated clusters can still form long-range interactions with other saturated or unsaturated clusters. After chromatin binding, binders were then allowed to form or break bonds with binders occupying neighbouring nodes in 3D space with probabilities (*k*_join_ and *k*_break_). Simulations were run for 5,000 time steps in 50–100 iterations. Model parameters are indicated in Supplementary Table 3. The parameters were changed as indicated for each model. In addition, in the capping model, each Ph-ML that joined a cluster removed one interaction surface from an existing Ph molecule in the cluster, such that the association rate for Ph of either type to a cluster with equal numbers of Ph and Ph-ML molecules went to zero.

## Additional information

**Accession codes:** Data sets generated for this work are archived in NCBI's Gene Expression Omnibus and are accessible through GEO Series accession numbers GSE60686, GSE61115, and GSE72830.

**How to cite this article**: Wani, A. H. *et al*. Chromatin topology is coupled to Polycomb Group protein subnuclear organization. *Nat. Commun.* 7:10291 doi: 10.1038/ncomms10291 (2016).

## Supplementary Material

Supplementary Information Supplementary Figures 1-12, Supplementary Tables 1-3 and Supplementary References
