# Supplementary material for: Chromatin topology is coupled to Polycomb group protein subnuclear organization
Source: Nat Commun. 2016 Jan 13;7:10291. doi: 10.1038/ncomms10291 (PMC4735512; doi:10.1038/ncomms10291)
Supplement: Supplementary Information — Supplementary Figures 1-12, Supplementary Tables 1-3 and Supplementary References [file ncomms10291-s1.pdf]

Supplementary Figure 1

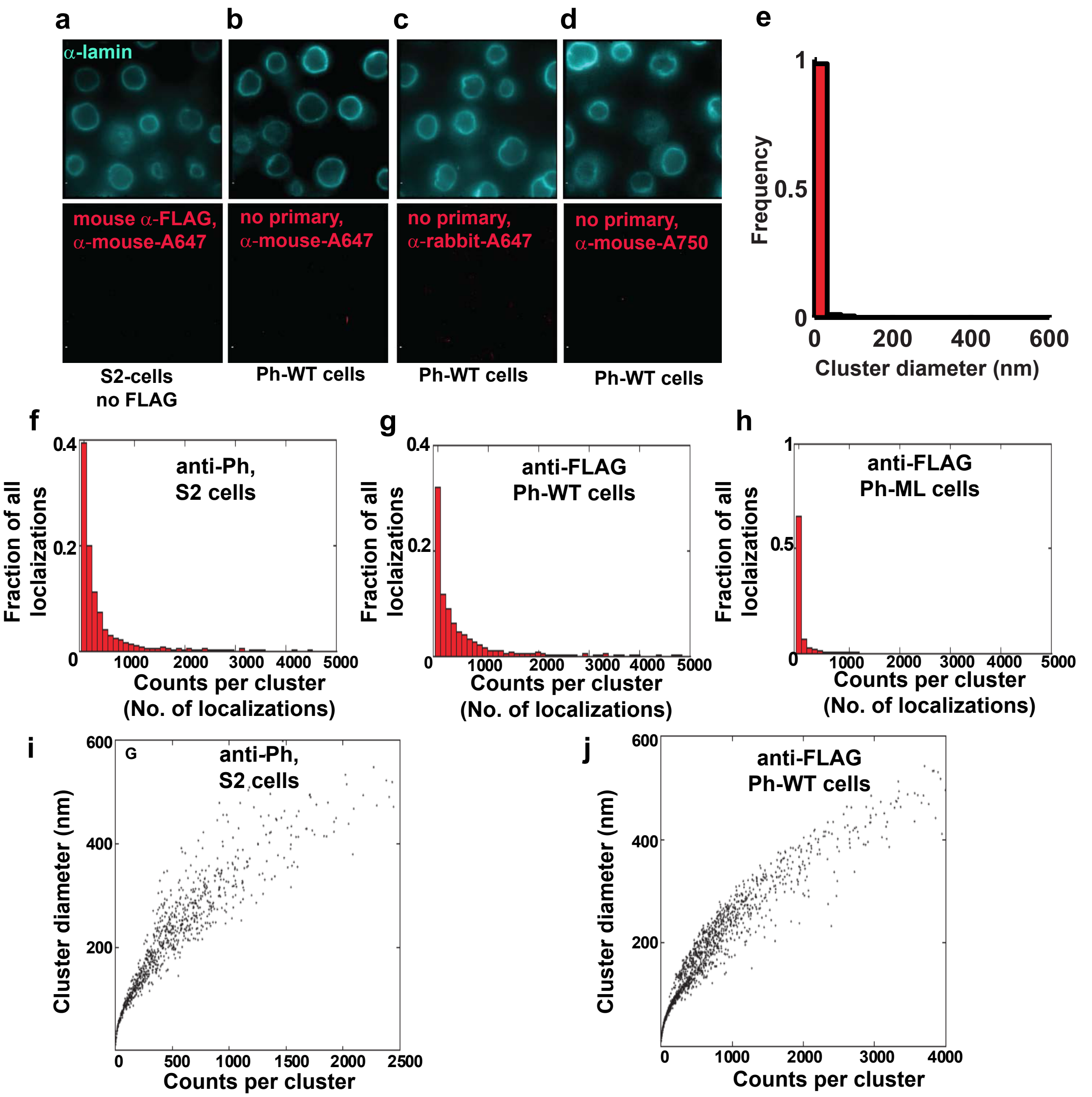

Supplementary Figure 1 | Immunofluorescence controls and localizations per cluster for STORM analysis. a-d. Images show representative results of control experiments for immunofluorescence. Top panels show immunofluorescence with anti-Lamin antibodies conjugated with Alexa-488 to visualize the outline of nuclei. Bottom panels show STORM images of same cells, which were immunostained as described next. **a.** Control showing lack of substantial localizations or localization clusters when the anti-FLAG antibody is used in S2 cells not expressing FLAG-tagged constructs. **b-d.** Controls showing lack of substantial localizations or localization clusters when the primary antibody is omitted from anti-mouse-647 (b), anti-rabbit-647 (c) and anti-mouse-750 (d) immunofluorescence experiments. **e.** Quantification of cluster-size distribution for control experiments shown in (a)-(d). Cells contained 1 ± 1 (mean ± std) cluster larger than the 30 nm resolution limit. Clusters smaller than 30 nm likely represent localizations from individual antibodies trapped non-specifically in the nucleus. **f-h.** Histograms show the distribution of the number of STORM localizations for the indicated target found in each cluster. The data are normalized by the total number of localizations. Individual dyes blink an average of 10 times, so total localizations should not be equated with molecule counts. **f.** Ph localizations in S2 cells. g. FLAG localization in cells expressing Ph-WT. **h.** FLAG localizations in cells expressing Ph-ML. **i,j.** Scatter plots showing the relationship between counts per cluster and cluster diameter for Ph clusters detected using anti-Ph antibody in S2 (h) or anti-FLAG antibody in Ph-WT cells. Note that the total counts are higher for clusters of the same diameter when anti-FLAG is used in place of anti-Ph. This could reflect differences in antibody binding efficiency and/or small differences in dye labeling of the secondary antibodies.

Supplementary Figure 2

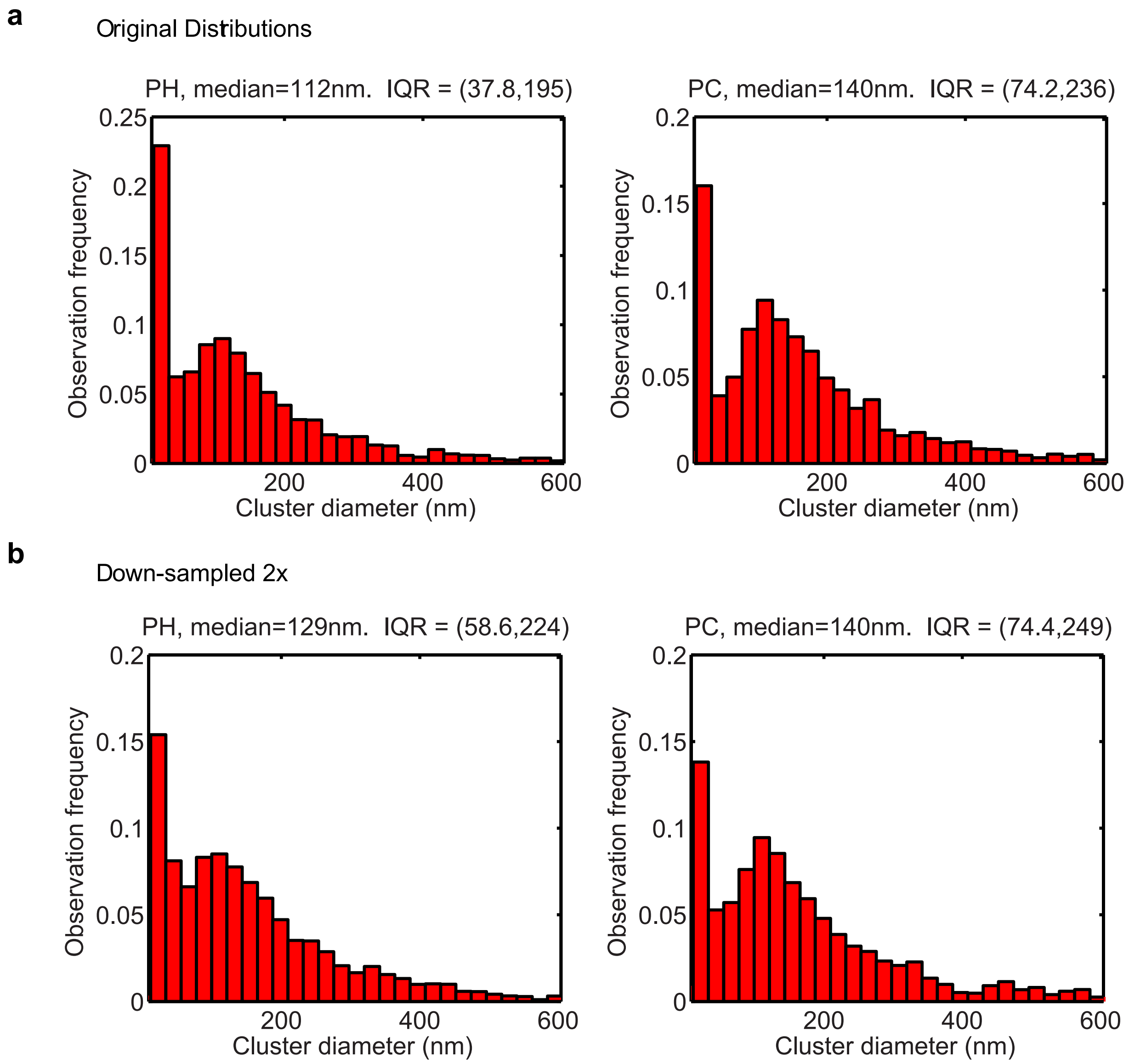

Supplemental Figure 2 | Effect of Downsampling Total Localizations. a. Distribution of cluster diameters reproduced from Figure 1. **b.** as in (a) but produced after removing a randomly selected 50% of the total localizations recorded. The median and inter-quatile range (IQR) are shown above each distribution.

Supplementary Figure 3

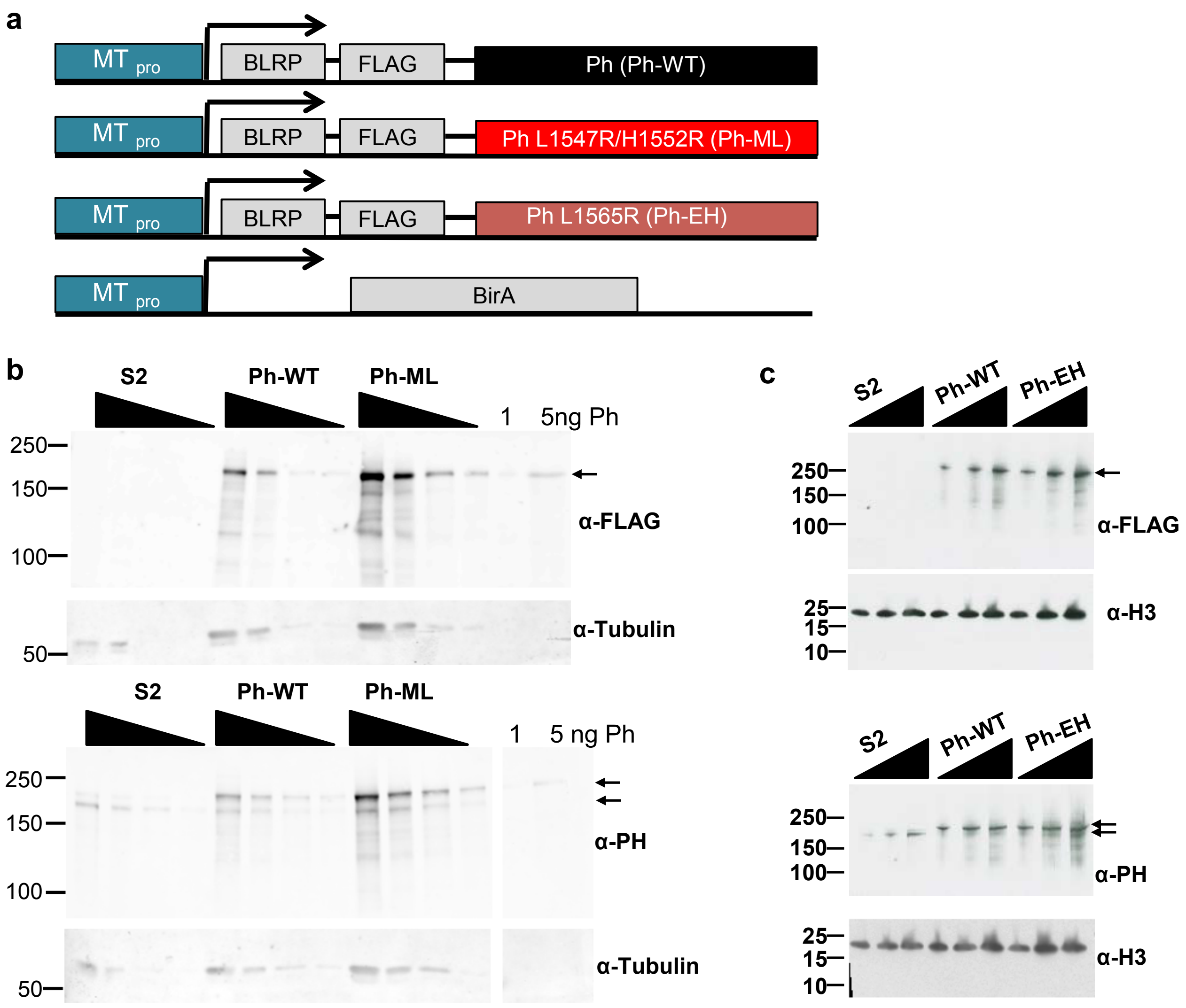

Supplementary Figure 3 | Stable *Drosophila* S2 cell lines expressing wild-type Ph (Ph-WT) or SAM polymerization defective Ph. a. Schematics showing constructs used. Cells were co-transfected with plasmids expressing wild type or mutant Ph that is tandem epitope tagged with the biotin ligase recognition peptide (BLRP) and FLAG, and the biotin ligase BirA, both under control of the metallothionin promoter (MT pro). Stable lines were selected with puromycin. Protein expression was induced by treatment of cells with 0.5 mM copper sulfate for three days. Ph-ML has the indicated mutations in the "mid-loop" polymerization interface of the SAM [23], and Ph-EH has the indicated mutation in the "end-helix" interface. **b,c.** Western blots of expression of Ph-WT and Ph-ML (b), or Ph-WT and Ph-EH (c). Arrows in anti-FLAG panels indicate the position of FLAG-tagged Ph, which is not present in control S2 cells. Arrows in the anti-Ph panels indicate ectopic (top arrow) and endogenous Ph. Note that there is more slowly migrating form of Ph also visible in the S2 cells.

Supplemental Figure 4

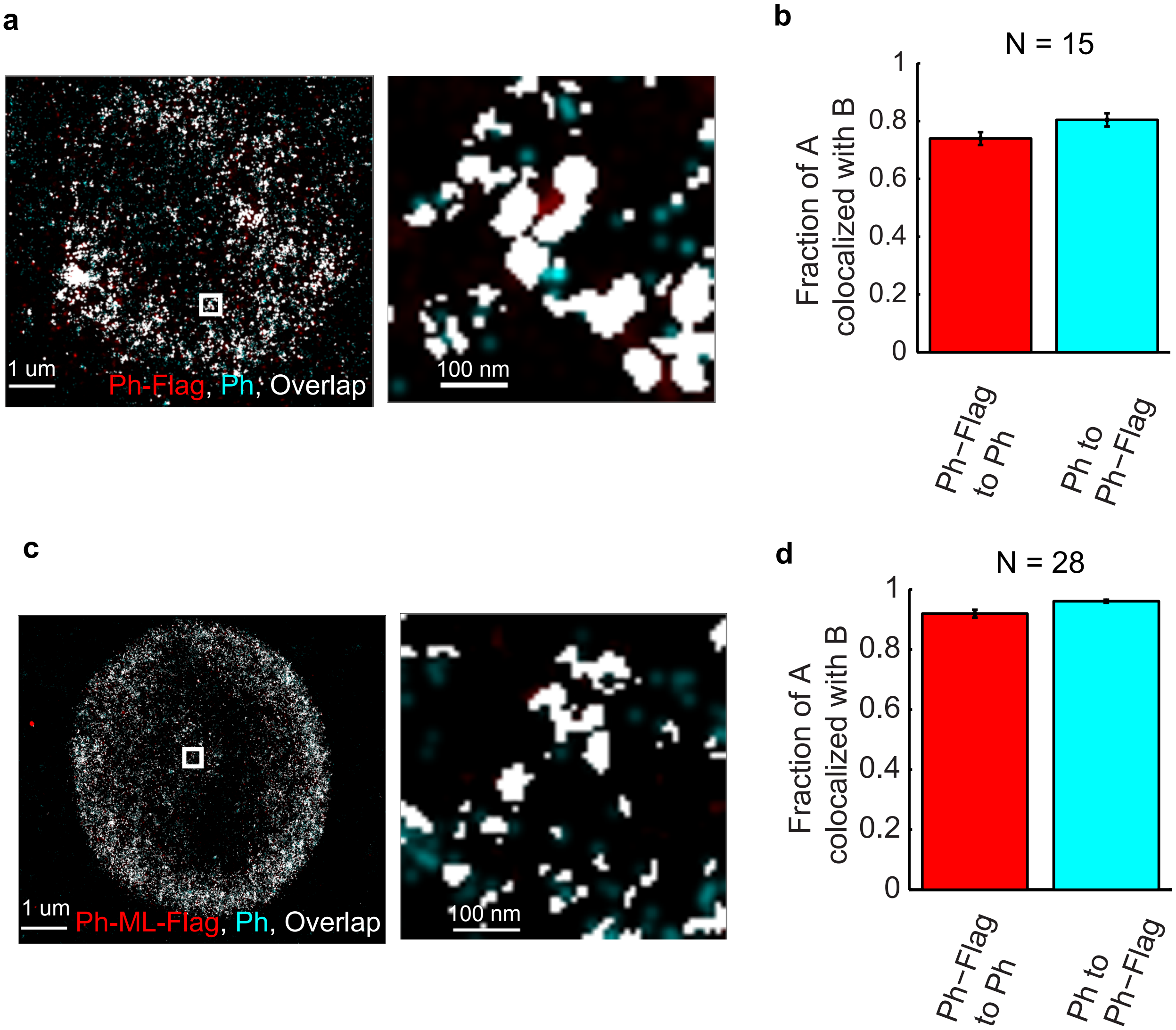

Supplemental Figure 4| Colocalization of Ph-Flag and Ph antibodies. a, Representative image of a PH-WT cell with clusters labeled by anti-FLAG (red), anti-Ph (cyan) or both (white). The region in the white box is shown in the blow-up to the right. **b**, Quantification from 15 Ph-WT cells of the fraction of localizations detected from antibodies against FLAG that co-clustered with localizations coming from antibodies against Ph (red) and vice versa (cyan). **c**, As in (a) but for Ph-ML-FLAG cells. **d**, as in (b) but for Ph-ML-FLAG cells.

Supplementary Figure 5

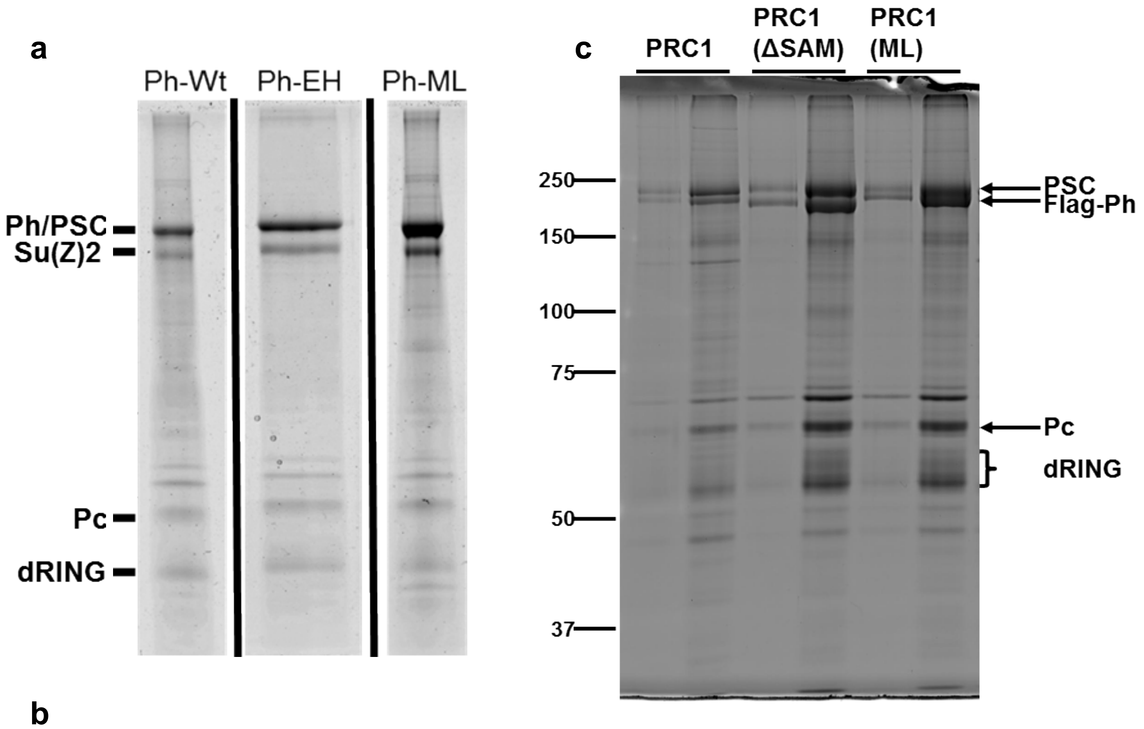

b

	Ph-WT	Ph-EH	Ph-ML
Polyhomeotic proximal (Ph-P)	87	84	70
Polyhomeotic distal (Ph-D)	11	10	8
Posterior Sex Combs (PSC)	150	128	111
Suppressor two of Zeste (Su(Z)2)	112	127	117
Polycomb (Pc)	42	54	50
dRING	42	38	46
Sex Comb on the Midleg (SCM)	64	12	1

Supplementary Figure 5 | PRC1 formation does not depend on Ph SAM polymerization activity. a. SAM polymerization defective Ph assembles into PRC1 in S2 cells. Image shows lanes from a colloidal blue stained SDS-PAGE gel of complexes purified from nuclear extracts of S2 cell lines expressing Ph-WT or Ph-ML. Complexes were purified with a two-step affinity purification protocol (anti-FLAG followed by streptavidin to capture the biotinylated BLRP tag). Note that the complexes used for glycerol gradient analysis (Fig. 2) were purified only with anti-FLAG. **b.** The identity of the core PRC1 subunits was confirmed by mass spectrometry. Su(Z)2 is a homologue of PSC [24]. We also identified SCM, a SAM containing PcG protein that associates at low levels with PRC1 via interactions with Ph SAM. SCM is reduced in Ph–ML purifications, as expected [25,26]. **c.** PRC1 with Ph-WT, Ph-ML, or Ph- delta SAM were reconstituted in Sf9 cells. Gel shows complexes purified from nuclei by anti-FLAG affinity chromatography and stained with SYPRO ruby.

Supplementary Figure 6

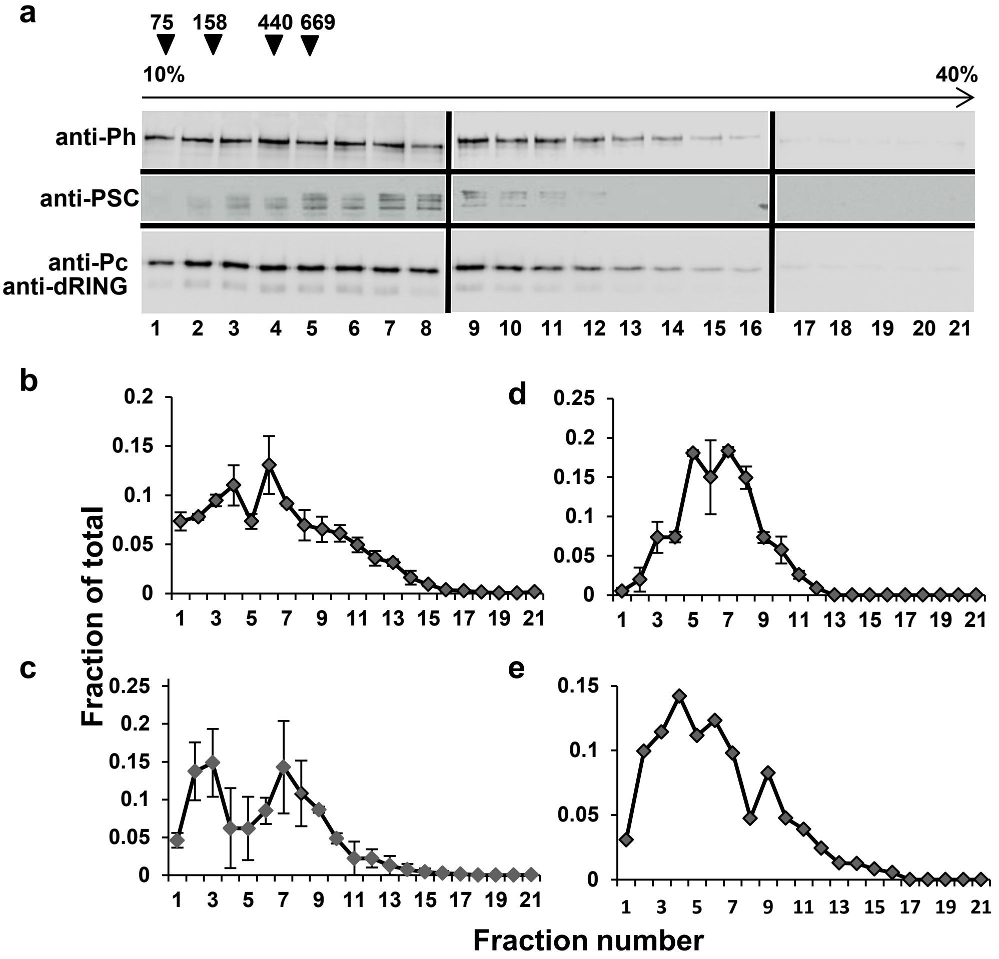

Supplementary Figure 6 | Sedimentation profile of complexes purified from nuclear extracts expressing Ph-EH. a. Western blots of gradient fractions of Ph-associated proteins purified from cells expressing Ph-EH. This gradient is directly comparable to the ones shown in Fig. 2**j**. **b-e**. Quantitation of western blots from gradients of Ph-EH associated proteins. Points show the average of gradients from two different preparations, except for (e) where only one replicate is plotted because the blot of the second one was not of sufficient quality for quantification; error bars represent the spread in the data. **b**. anti-Ph, **c**. anti-PSC, **d**. anti-Pc, **e**. anti-dRING.

Supplementary Figure 7

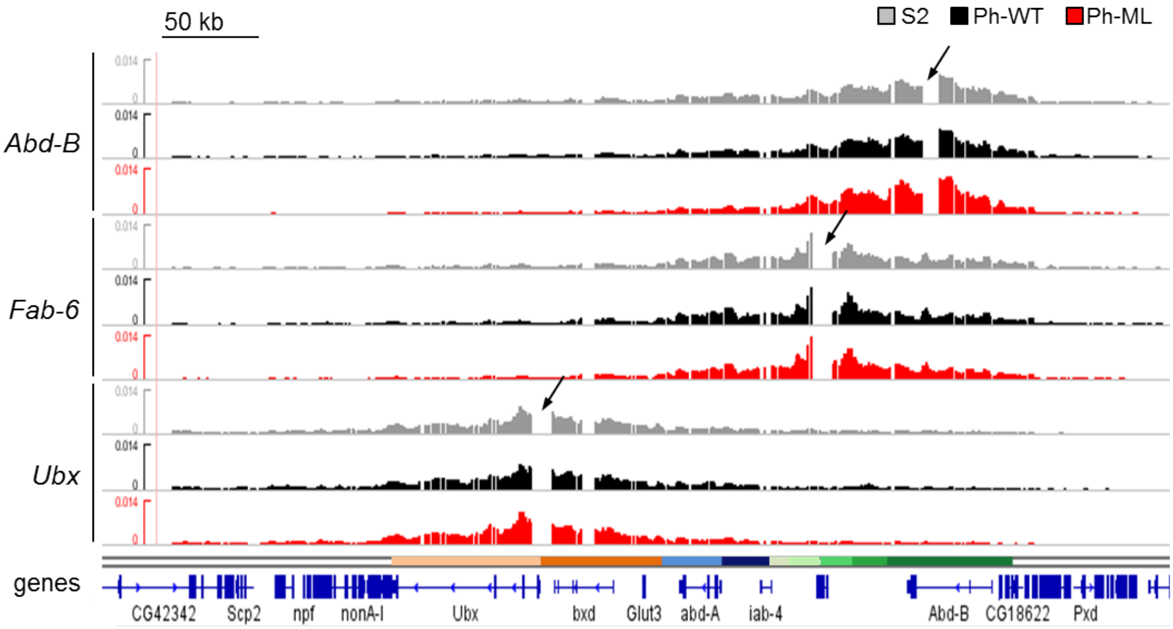

Supplementary Figure 7 | 4C-seq profiles of *BX-C*. 4C-seq contacts in the *BX-C* region were mapped using the HTS pipeline and normalized by the respective total number of contacts in the *BX-C* for each cell type and viewpoint. Traces show the average of two experiments. Baits are indicated with arrows. A 10 kb region surrounding the bait sequence was excluded from the analysis. Coloured track indicates *BX-C* regulatory regions (as in Fig. 3a) (green regions regulate *Abd-B*, blue regulate *abd-A*, and orange regulate *Ubx*).

Supplementary Figure 8

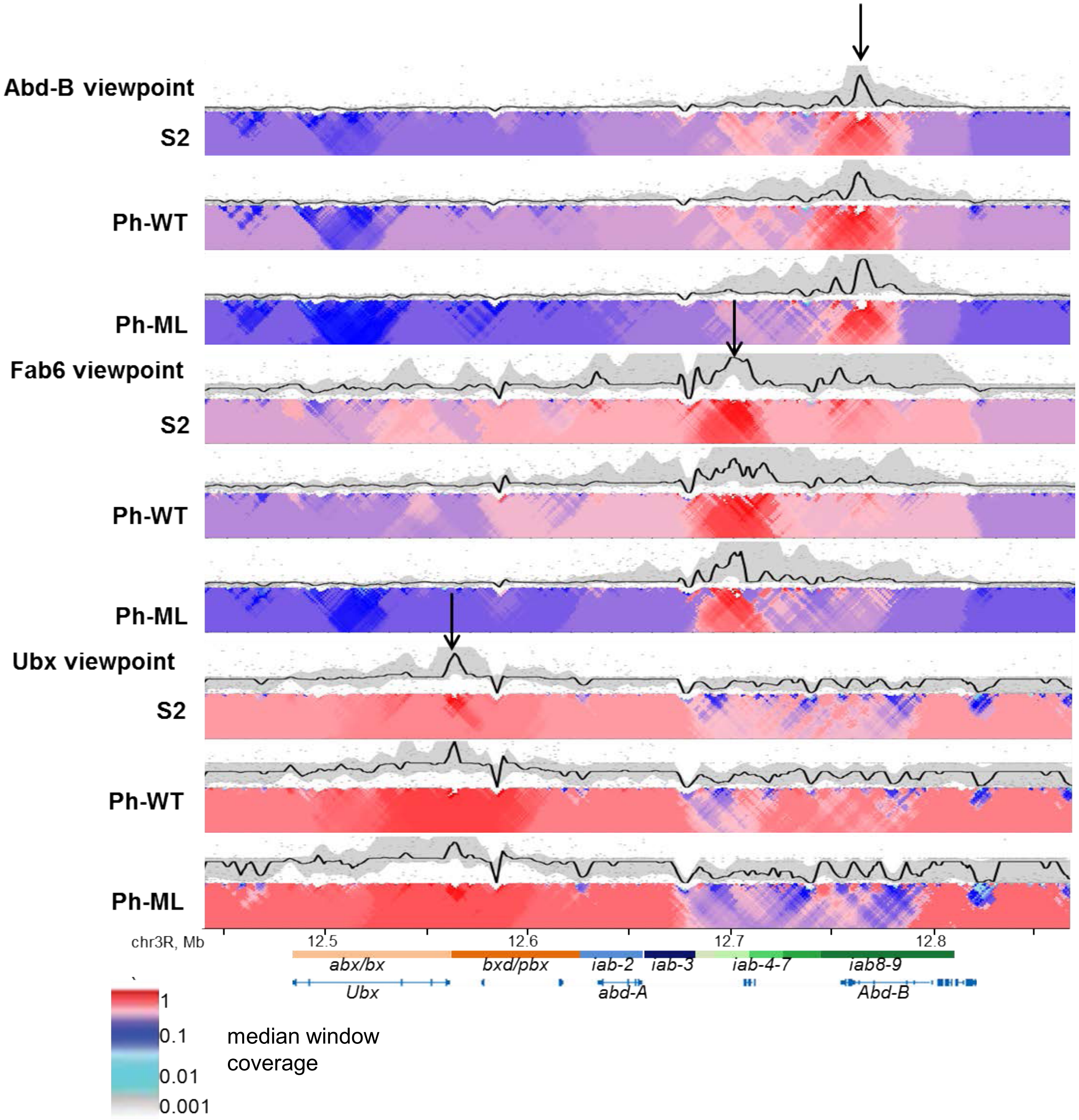

Supplementary Figure 8 | 4C-seqpipe profiles of the BX-C. To visualize specific contacts inside the BX-C, we used 4c-seqpipe [10] to generate normalized plots of the BX-C. Data are shown for one of the two replicates for each viewpoint for S2, Ph-WT, and Ph-ML cells. The data show that specific contacts are made between the viewpoints and sequences in the BX-C, and these contacts are reduced in Ph-ML cells. We do not find evidence for different contacts being made in Ph-ML cells. The trendline is the median and was plotted using a 5kb window. Grey shading indicates the interquartile range (20th to 80th percentile). The domaingram scale is from 2 to 50 kb.

Supplementary Figure 9

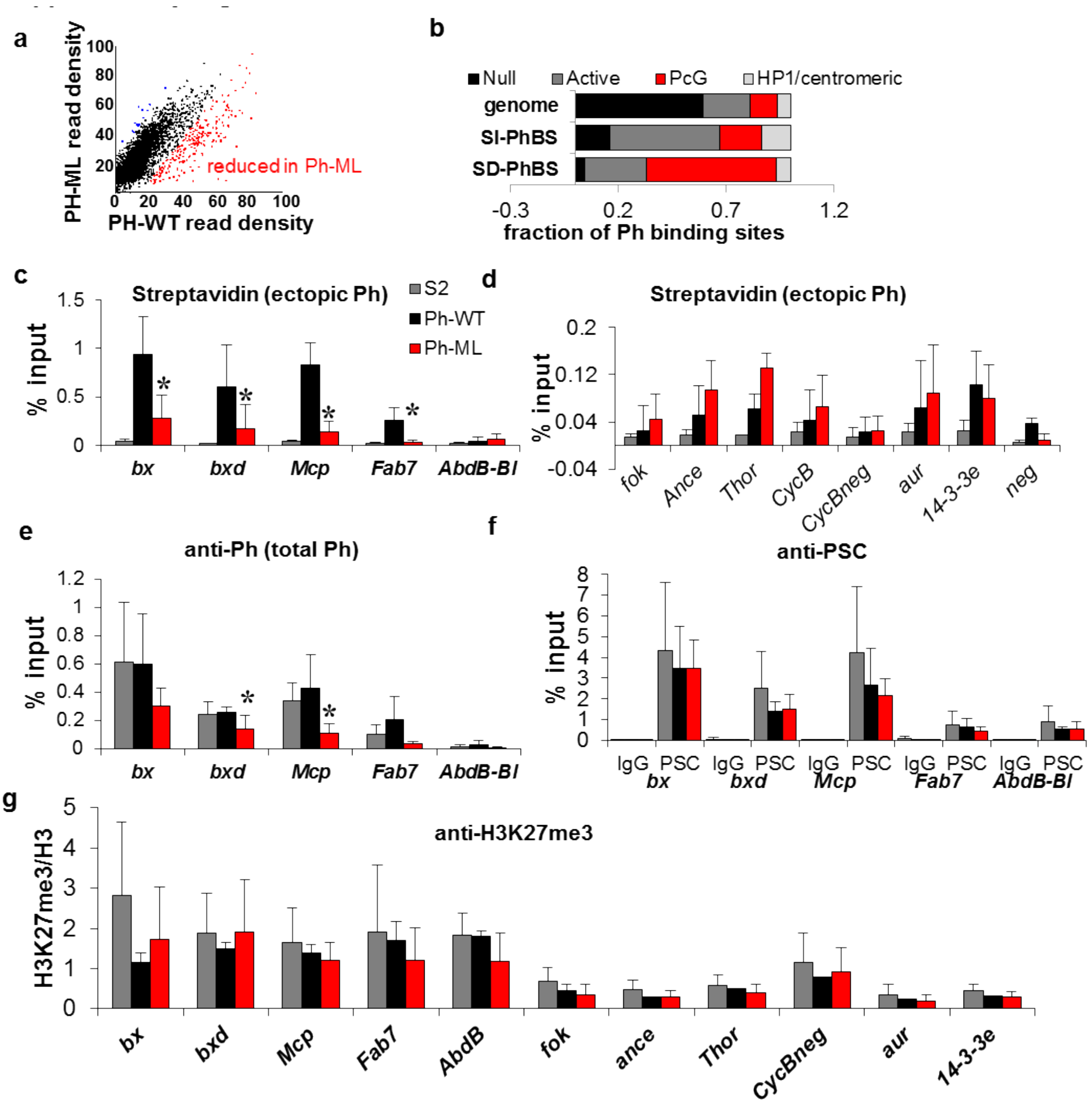

Supplementary Figure 9 | Genome wide analysis of Ph and Ph-ML binding to chromatin. a. Scatter plot showing all Ph binding sites in Ph-WT and Ph-ML expressing cells. **b**. Distribution of Ph-ML peaks that are unchanged (SAM-independent (SI)) or have decreased binding (SAM-dependent, SD) over different classes of chromatin topological domains (as defined in 27). Domains were normalized by size in base pairs. **c-g**. ChIP-qPCR analysis of Ph binding sites. **c,d**. Streptavidin pulldown of ectopically expressed Ph-WT or Ph-ML at SD-PhBS (c) and SI-PhBS (d). AbdB-BI is inside the BX-C but is a SI-PhBS. “neg” and “CycBneg” sites are not expected to bind PcG proteins and the S2 sample does not express a biotin-tagged protein. **e**. ChIP with antibodies against total Ph. **f**. ChIP with antibodies to the PRC1 component PSC. **g**. ChIP with antibodies against the PcG associated histone modification H3K27me3. Bars show the average of at least 4 experiments, except for panel g, which is the average of two experiments. Error bars are the standard deviation, and asterisks indicate significant differences ($p \leq 0.05$) between Ph-ML and Ph-WT cells as determined by paired, two-tailed student’s t-test. Legend for d-g is the same as in c.

Supplementary Figure 10

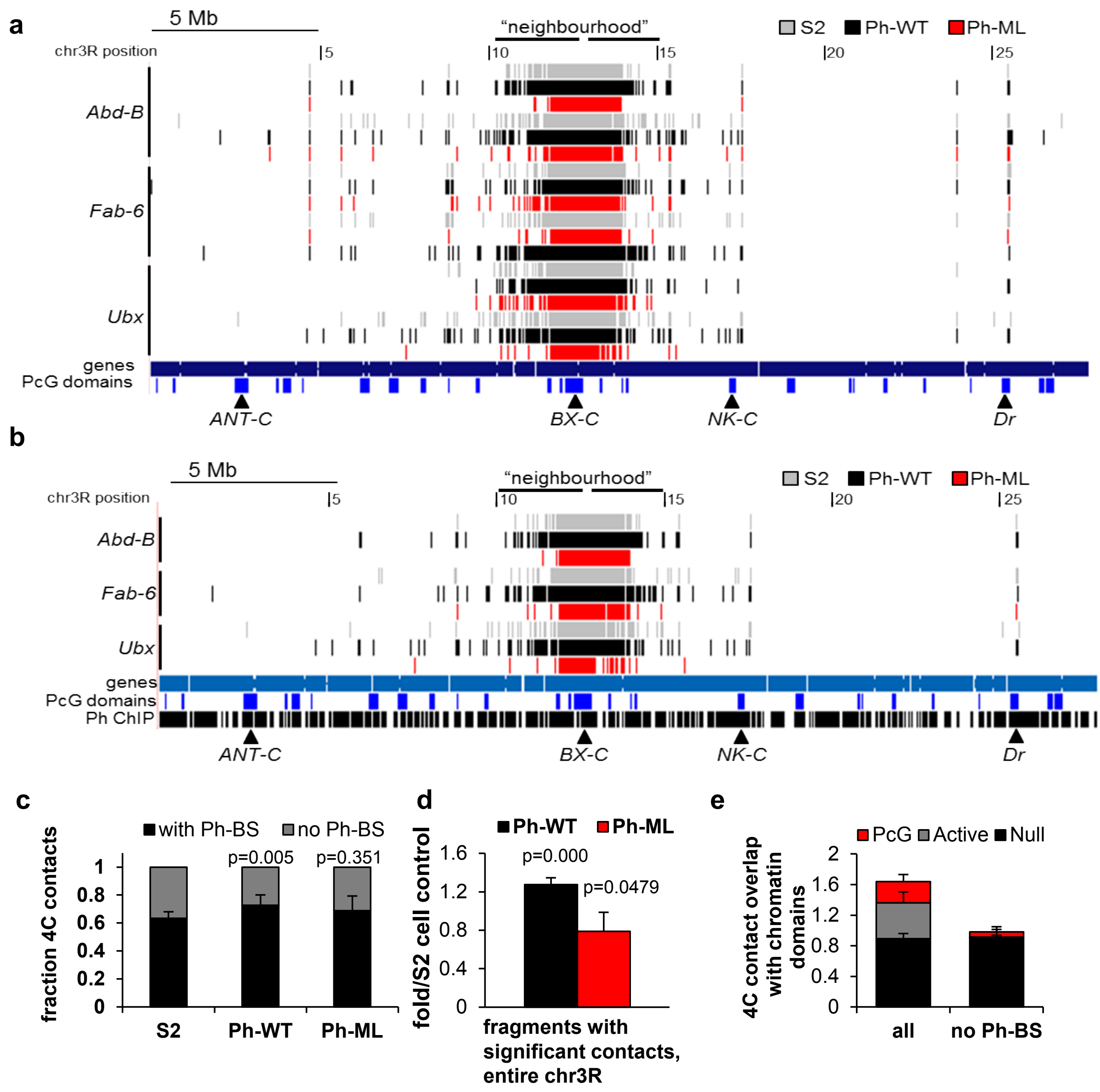

Supplementary Figure 10 | *fourSig* analysis of Chromosome 3R. a. Traces show significant contacts identified on Chromosome 3R using *fourSig* for both replicates of the 4C-seq experiment. **b.** Traces show significant contacts identified on Chromosome 3R using *fourSig* that also overlap Ph binding sites. **c**. Graph shows the average fraction of 4C contacts that overlap at least one Ph binding site. p-values are for paired, 2-tailed student's t-test of Ph-WT and S2 cell data. **d.** Comparison of the number of fragments in significant contacts calculated over Chromosome 3R. Each data set was normalized to its corresponding S2 cell control. Bars show the average of these ratios and error bars are the standard deviation. p-values are for the difference from the expected ratio of 1 by 1-sample, 2-tailed t-test. **e.** Fraction of contacts that overlap different types of chromatin domains, segregated by whether they also overlap at least one Ph peak. Note that many contacts overlap more than one domain. p-values are for paired, 2-tailed student's t-test of all contacts versus those that do not overlap any Ph-BS.

Supplementary Figure 11

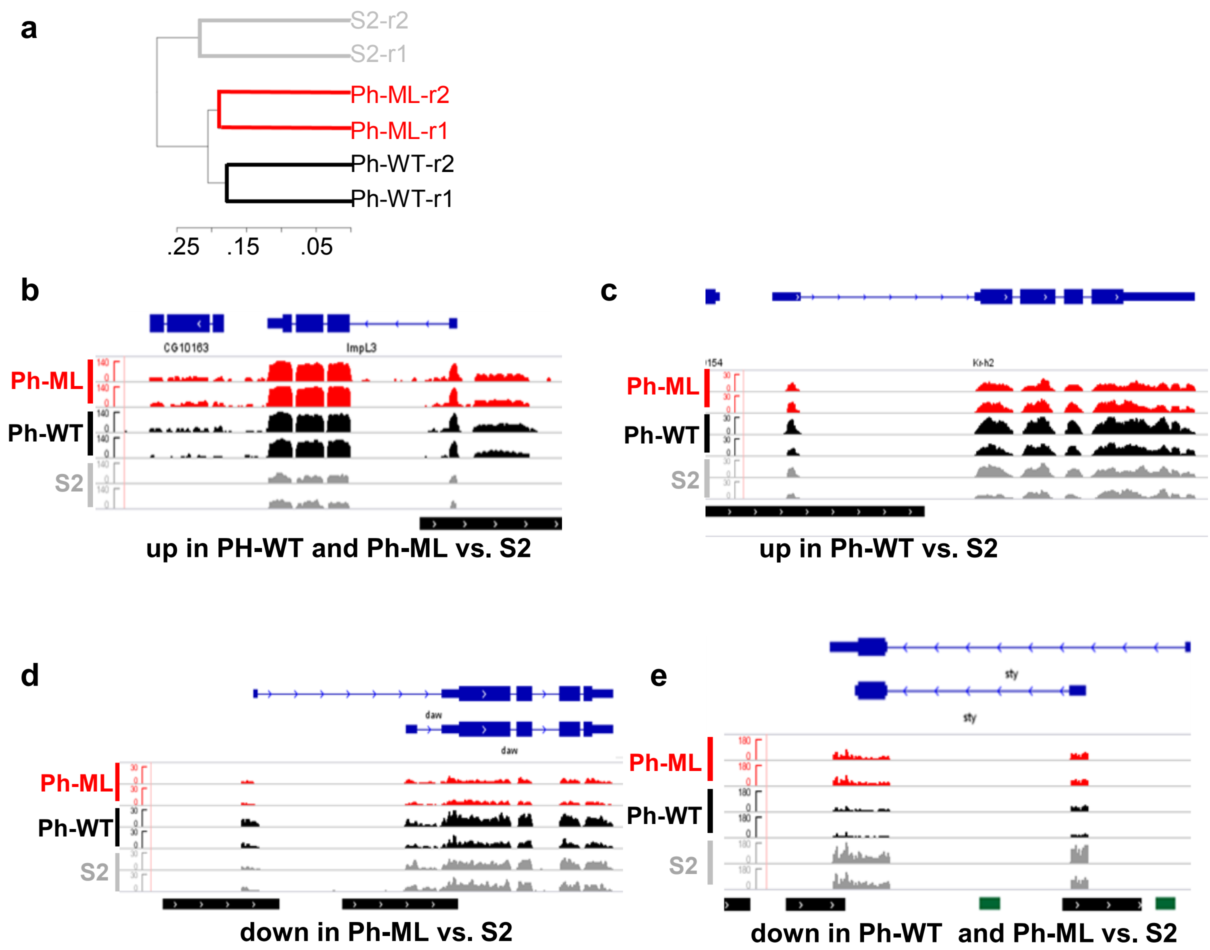

Supplementary Figure 11 | Transcriptome analysis of Ph-WT, Ph-ML, and S2 cells by RNA-seq. a. Dendrogram of RNA-seq replicates indicating that Ph-ML and Ph-WT transcriptomes are more similar to each other than to S2 cells, and Ph-ML cells are more similar to S2 cells than are Ph-WT. This is consistent with the finding that many changes in gene expression are shared between the two cell lines. **b-e.** Examples of classes of genes whose expression levels are changed in Ph-ML and/or Ph-WT cells. Traces are as in Fig. 4a-b, but show examples of each of the other classes of genes identified.

Supplementary Figure 12

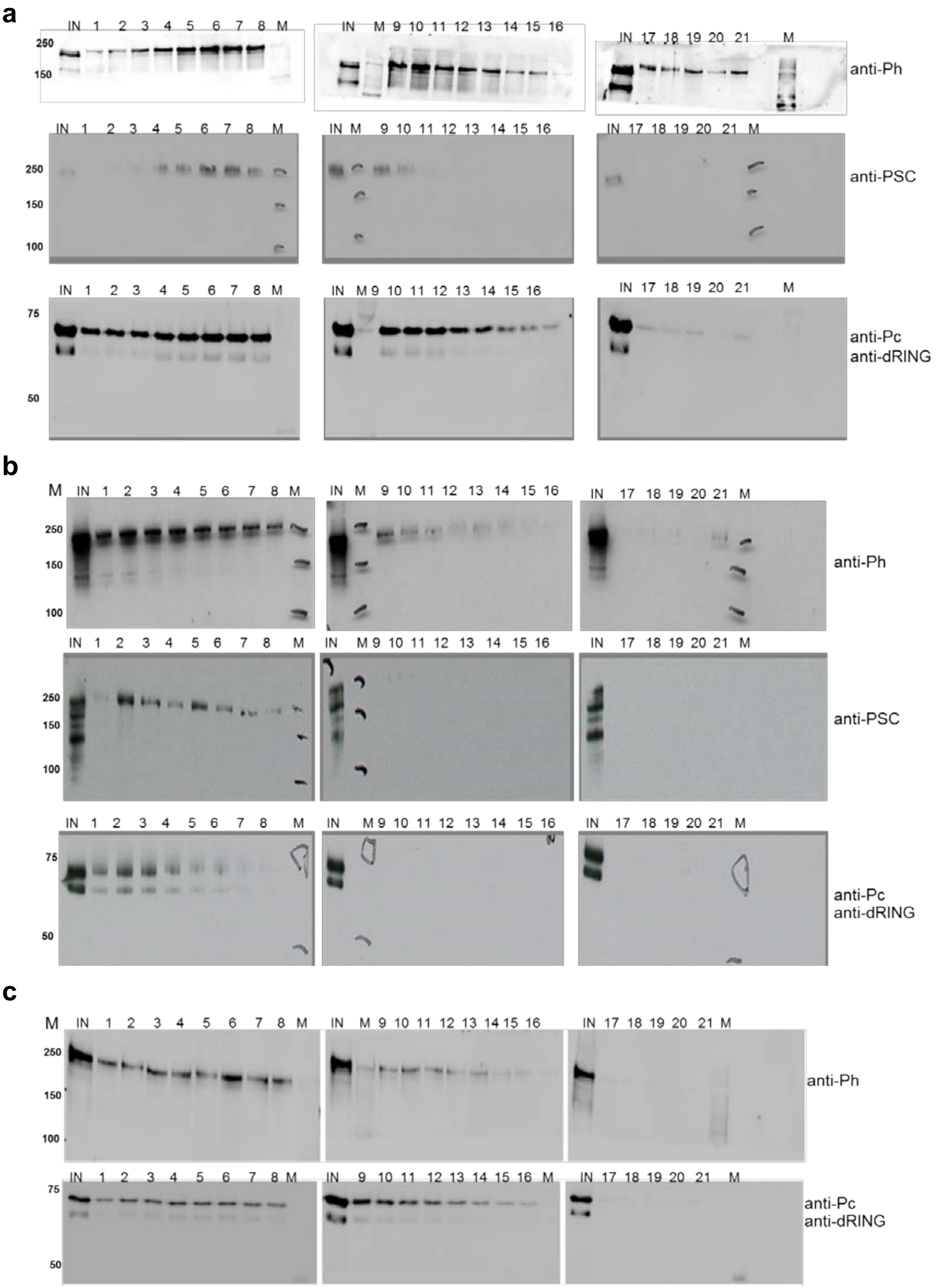

Supplementary Figure 12 | Sedimentation profile of complexes purified from nuclear extracts expressing Ph-WT, Ph-ML or Ph-EH. Images show full Western blots used to create the images in Supplementary Figure 6a and Figure 2j. M=molecular weight marker; I=input (loaded on each gel to use as a standard for quantification). Fraction numbers are indicated on each lane. Note that blots were cut above the 75 kDa marker so that the top was probed with anti-Ph and the bottom with anti-Pc and anti-dRING. **a.** Ph-WT. **b.** Ph-ML. **c.** Ph-EH.

Supplementary Table 1 ChIP-qPCR primers

**Site**	**Forward**	**Reverse**
bx	TAGTCTTATCTGTATCTCGCTCTTA	CAGAACCAAAGTGCCGATAACTC
bxd	CCATAAGAAATGCCACTTTGC	CTCTCACTCTCTCACTGTGAT
Fab7	GGAATACCGCACTGTCGTAGG	GCAGCCATCATGGATGTGAA
mcp	AGATGGCAACATTGGCTGC	CGGATCTGCAGTCAAACGTCAC
Abd-B-Bl	TTTCCACATTCCCGCCTTGCAG	ATGTCCACTGTCGGAGAGCGAC

Supplementary Table 2 Primers for 4C-seq

**Viewpoint**	**Left inverse primer**	**Barcode**
Ubx	AATGATACGGCGACCACCGAACACTCTTTCCCTACACGACGCTCTTCCGATCT**CGTGAT**CCAACTCGGCATTGTACGGTAA	1
	AATGATACGGCGACCACCGAACACTCTTTCCCTACACGACGCTCTTCCGATCT**ACATCG**CCAACTCGGCATTGTACGGTAA	2
	AATGATACGGCGACCACCGAACACTCTTTCCCTACACGACGCTCTTCCGATCT**GCCTAA**CCAACTCGGCATTGTACGGTAA	3
Abd-B	AATGATACGGCGACCACCGAACACTCTTTCCCTACACGACGCTCTTCCGATCT**CGTGAT** TGGTGGATGAACTCCCAAAAA	1
	AATGATACGGCGACCACCGAACACTCTTTCCCTACACGACGCTCTTCCGATCT**ACATCG** TGGTGGATGAACTCCCAAAAA	2
	AATGATACGGCGACCACCGAACACTCTTTCCCTACACGACGCTCTTCCGATCT**GCCTAA** TGGTGGATGAACTCCCAAAAA	3
Fab-6	AATGATACGGCGACCACCGAACACTCTTTCCCTACACGACGCTCTTCCGATCT**CGTGAT**CGTTGATGGTTCGGGGTATTATCT	1
	AATGATACGGCGACCACCGAACACTCTTTCCCTACACGACGCTCTTCCGATCT**ACATCG**CGTTGATGGTTCGGGGTATTATCT	2
	AATGATACGGCGACCACCGAACACTCTTTCCCTACACGACGCTCTTCCGATCT**GCCTAA**CGTTGATGGTTCGGGGTATTATCT	3
**Viewpoint**	**Right inverse primer**	
Ubx	CAAGCAGAAGACGGCATACGAAGGAAAATCAGCCCTCCTCCAT	
	CAAGCAGAAGACGGCATACGAAGGAAAATCAGCCCTCCTCCAT	
	CAAGCAGAAGACGGCATACGAAGGAAAATCAGCCCTCCTCCAT	
Abd-B	CAAGCAGAAGACGGCATACGACACTCGGAGCCACTTGCAT	
	CAAGCAGAAGACGGCATACGACACTCGGAGCCACTTGCAT	
	CAAGCAGAAGACGGCATACGACACTCGGAGCCACTTGCAT	
Fab-6	CAAGCAGAAGACGGCATACGAAGAGTTGCTCGGCGAGTG	
	CAAGCAGAAGACGGCATACGAAGAGTTGCTCGGCGAGTG	
	CAAGCAGAAGACGGCATACGAAGAGTTGCTCGGCGAGTG	

*Barcodes 1, 2 and 3 (bold) were used for *Drosophila* S2 cells, cells expressing Ph-WT and cells expressing Ph-ML, respectively. Underlined sequences are specific to the viewpoint; additional sequences are adaptors for sequencing.

Supplementary Table 3 Model parameters

**Model \ Parameters**	$N_{endog.}$	$k_a^{endog.}$	$k_d^{endog.}$	$k_a^{exog.}$	$k_d^{exog.}$	k_{join}	k_{break}	E_{bond}	$N_{exog.}$	A_{node}	N_{max}
**Low-affinity**	2000	0.0008	0.05	0.00016	0.25	0.75	0.01	0.75	0-3000	B(0.4)	15
**Capping**	2000	0.0008	0.05	0.0008	0.05	0.75	0.01	0.75	0-3000	B(0.4)	15
**Unlimited spreading**	2000	0.0008	0.05	0.0008	0.05	0.75	0.01	0.75	0-2000	N(0.3,1)	Inf.
**Limited spreading**	2000	0.0008	0.05	0.0008	0.05	0.75	0.01	0.75	0-2000	N(0.3,1)	15

$N_{endog.}$ – Number of endogenous Ph molecules in the simulation.

$k_a^{endog.}$ – Association rate constant between a bound Ph molecule and a freely diffusing Ph molecule.

$k_d^{endog.}$ – Dissociation rate constant for a bound Ph molecule.

$k_a^{exog.}$ – Association rate constant between a bound Ph molecule and a freely diffusing Ph-ML molecule.

$k_d^{exog.}$ – Dissociation rate constant for a bound Ph-ML molecule.

k_{join} – Probability per time step that two adjacent clusters form a bond.

k_{break} – Probability per time step that two bound clusters break their bond.

E_{bond} – bond energy between adjacent clusters that determines the probability that clusters dissociate during a proposed chain move.

$N_{exog.}$ – Number of exogenous Ph molecules (Ph-ML or Ph-WT) in the simulation.

A_{node} – Binding affinity of nodes – a random number between [0,1] which modulates the probability of binding at a particular site. *B(x)* indicates Bernoulli random variable, = 1 with probability *x*, 0 with probability 1-*x*. *N(x,y)* indicates a random number drawn from a Normal distribution of mean x and standard deviation *y*, and restricted to the interval [0,1].

N_{max} – Maximum number of molecules which can bind a individual node on the polymer

Supplementary References:

1 Kim, C. A., Gingery, M., Pilpa, R. M. & Bowie, J. U. The SAM domain of polyhomeotic forms a helical polymer. *Nature structural biology* **9**, 453-457 (2002).

2 Lo, S. M., Ahuja, N. K. & Francis, N. J. Polycomb group protein Suppressor 2 of zeste is a functional homolog of Posterior Sex Combs. *Molecular and cellular biology* **29**, 515-525 (2009).

3 Kim, C. A., Sawaya, M. R., Cascio, D., Kim, W. & Bowie, J. U. Structural organization of a Sex-comb-on-midleg/polyhomeotic copolymer. *The Journal of biological chemistry* **280**, 27769-27775 (2005).

4 Peterson, A. J., Mallin, D. R., Francis, N. J., Ketel, C. S., Stamm, J., Voeller, R. K., Kingston, R. E. & Simon, J. A. Requirement for sex comb on midleg protein interactions in Drosophila polycomb group repression. *Genetics* **167**, 1225-1239 (2004).

5 van de Werken, H. J., Landan, G., Holwerda, S. J., Hoichman, M., Klous, P., Chachik, R., Splinter, E., Valdes-Quezada, C., Oz, Y., Bouwman, B. A., Verstegen, M. J., de Wit, E., Tanay, A. & de Laat, W. Robust 4C-seq data analysis to screen for regulatory DNA interactions. *Nature methods* **9**, 969-972 (2012).

6 Sexton, T., Yaffe, E., Kenigsberg, E., Bantignies, F., Leblanc, B., Hoichman, M., Parrinello, H., Tanay, A. & Cavalli, G. Three-dimensional folding and functional organization principles of the Drosophila genome. *Cell* **148**, 458-472 (2012).
